# Best emollients for eczema (BEE) – comparing four types of emollients in children with eczema: protocol for randomised trial and nested qualitative study

**DOI:** 10.1136/bmjopen-2019-033387

**Published:** 2019-11-06

**Authors:** Matthew J Ridd, Sian Wells, Louisa Edwards, Miriam Santer, Stephanie MacNeill, Emily Sanderson, Eileen Sutton, Alison R G Shaw, Jonathan Banks, Kirsty Garfield, Amanda Roberts, Tiffany J Barrett, Helen Baxter, Jodi Taylor, J Athene Lane, Alastair D Hay, Hywel C Williams, Kim Suzanne Thomas

**Affiliations:** 1 Centre for Academic Primary Care, Bristol Medical School, University of Bristol, Bristol, UK; 2 Faculty of Health Sciences, Simon Fraser University, Burnaby, British Columbia, Canada; 3 Primary Care and Population Sciences, University of Southampton, Southampton, UK; 4 Bristol Randomised Trials Collaboration, Bristol Trials Centre, University of Bristol, Bristol, UK; 5 National Institute for Health Research Collaborations for Leadership in Applied Health Research and Care West (NIHR CLAHRC West), University Hospitals Bristol NHS Foundation Trust, Bristol, UK; 6 Nottingham Support Group for Carers of Children with Eczema, Nottingham, UK; 7 South West Medicines Information & Training, University Hospitals Bristol NHS Foundation Trust, Bristol, UK; 8 Centre of Evidence-Based Dermatology, University of Nottingham, Nottingham, UK

**Keywords:** eczema, paediatric dermatology, therapeutics

## Abstract

**Introduction:**

Atopic dermatitis/eczema affects around 20% of children and is characterised by inflamed, dry, itchy skin. Guidelines recommend ‘leave-on’ emollients that are applied directly to the skin to add or trap moisture and used regularly, they can soothe, enhance the skin barrier and may prevent disease ‘flares’. However, the suitability of the many different emollients varies between people and there is little evidence to help prescribers and parents and carers decide which type to try first.

**Methods and analysis:**

Design: pragmatic, multicentre, individually randomised, parallel group superiority trial of four types of emollient (lotions, creams, gel or ointments).

Setting: general practitioner surgeries in England.

Participants: children aged over 6 months and less than 12 years with mild-to-severe eczema and no known sensitivity to study emollients.

Interventions: study-approved lotion, cream, gel or ointment as the only leave-on emollient for 16 weeks, with directions to apply twice daily and as required. Other treatments, such as topical corticosteroids, used as standard care.

Follow-up: 52 weeks.

Primary outcome: validated patient-orientated eczema measure measured weekly for 16 weeks.

Secondary outcomes: eczema signs (Eczema Area Severity Index) by masked researcher, treatment use, parent satisfaction, adverse events, child and family quality of life (Atopic Dermatitis Quality of Life, Child Health Utility 9D and Dermatitis Family Impact).

Sample size: 520 participants (130 per group).

Analysis: intention-to-treat using linear mixed models for repeated measures.

Nested qualitative study: audio-recording of sample of baseline appointments and up to 60 interviews with participants at 4 and 16 weeks, interviews to be transcribed and analysed thematically.

**Ethics and dissemination:**

Ethics approval granted by the NHS REC (South West - Central Bristol Research Ethics Committee 17/SW/0089). Findings will be presented at conferences, published in open-access peer-reviewed journals and the study website; and summaries shared with key stakeholders.

**Trial registration number:**

ISRCTN84540529

**Strength and limitations of this study**

- First, adequately powered head-to-head pragmatic trial of the four main types of emollient prescribed for the treatment of eczema in children, recruited from primary care, with long-term follow-up.
- The primary core outcome is a validated patient-reported measure (POEM) that captures symptoms of eczema that matter to patients, and weekly measures over the 16 weeks mean that all participants who complete at least one POEM post-baseline will be included in the analysis. Researchers undertaking assessments of eczema signs (secondary outcome) are masked to allocation and use validated core outcome (Eczema Area Severity Index).
- Parents and their clinicians are unmasked and therefore their assessment of both the effectiveness and acceptability of the study emollient may be biased.
- Study emollients of each type are similar, increasing generalisability of the findings, but because they are not identical subtle differences both within-types and between-types may not be identified.
- The findings will reduce ‘trial-and-error’ prescribing of initial choice of emollient but should not be used to restrict emollient options.

## Introduction

### Background and rationale

Eczema affects around 20% of children.^1^ It is characterised by dry and inflamed itchy skin, and it can have a significant impact on the quality of life for both the child and their family.^2^ In accordance with the recommended nomenclature of the World Allergy Organisation, we use the label ‘eczema’ to refer to the clinical phenotype of atopic eczema/dermatitis.^3^

The majority of children with eczema have disease of mild or moderate severity and are diagnosed and managed exclusively in primary care.^4^ Children are commonly prescribed a moisturiser (emollient) and topical corticosteroid/topical calcineurin inhibitor to use alongside to treat or prevent ‘flares’.^5^ By direct application to the skin, emollients improve skin hydration and reduce symptoms such as stinging or itching, but they can also act as a barrier to potential irritants. Mild anti-inflammatory properties may reduce reliance on topical corticosteroids/calcineurin inhibitors.^6^ Many directly applied or ‘leave-on’ emollients can also be used as soap substitutes.

However, there are many different emollients available and little evidence that any one emollient is better than another as a leave-on treatment. The main formulations are lotions, creams, gels and ointments, which vary in their consistency from ‘light’ to ‘heavy’. This mainly reflects differences in their oil (lipid) to water ratios. Some products also contain humectants which help retain moisture, but emollients containing urea or antimicrobial compounds tend to be reserved for more severe disease.

The absence of evidence regarding the comparative clinical and cost-effectiveness of different products is reflected in emollient formularies. Clinician prescribing in the National Health Service (NHS) is guided by locally produced and maintained formularies, which recommend which items should be prescribed in that area. In 2018, across England and Wales there were over 100 different emollient formularies which made widely varying recommendations about 109 different emollients.^7^ The current situation where healthcare professionals recommend different emollients and carers find an effective emollient through a process of ‘trial and error’ is detrimental to both families and the NHS.^8 9^

In 2007, National Institute for health and Clinical Excellence (NICE) recommended research to identify ‘the most effective and cost-effective combinations of emollient products to use for the treatment of childhood atopic eczema’.^5^ A recently published Cochrane review identified 77 trials, comprising 6603 participants, evaluating the effectiveness of emollients.^6^ The authors were unable to conclude whether some of the moisturisers, or their ingredients, are better than others, and recommended head-to-head comparisons in clinical trials.

### Aim and objectives

The aim of the study is to compare the effectiveness and acceptability of four types of emollient (lotion, cream, gel and ointment) commonly used to treat eczema.

The objectives are to compare the four different emollient types, over the medium (16 weeks) and long-term (52 weeks), with respect to:

- Parent-reported eczema symptoms.
- Researcher assessment of eczema signs.
- Quality of life for the child.
- Impact of eczema on the family.
- Adverse effects.
- Acceptability of and parent satisfaction with study emollient.
- Frequency and quantity of study emollient and other emollient use.
- Use of other eczema treatments (including topical corticosteroid and calcineurin inhibitor).
- Number of well-controlled weeks.

### Trial design

Best emollients for eczema (BEE) is a pragmatic, multicentre, individually randomised, parallel group superiority trial of four types of emollient in children with eczema, with nested qualitative study.

It is a type A Clinical Trial of an Investigational Medicinal Product trial, which is low risk because the use of the medicinal product is not higher than the risk of standard medical care.

## Methods and analysis

### Study setting

Primary care (general practitioner (GP) surgeries) in and around Bristol, Southampton and Nottingham.

### Recruitment

**Figure 1:**
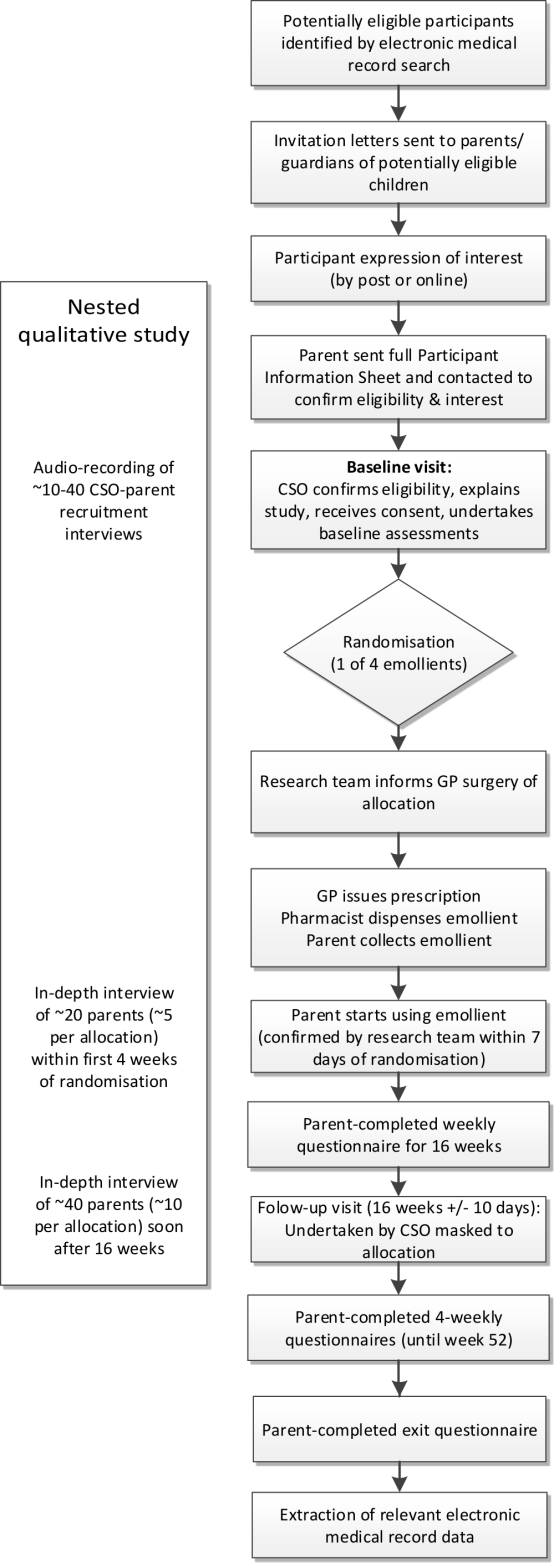
Overview of participant pathway through the study. GP, general practitioner.

The stages of participant recruitment are shown in figure 1.

We will identify children aged between 6 months and less than 12 years with eczema via an electronic medical records search. A GP or a delegated member of the practice team will screen the search results for inclusion/exclusion criteria. Parents and carers (hereafter parents) of potentially eligible children will be posted an invitation. In addition, GPs can recruit participants opportunistically.

Interested parents will complete a brief screening questionnaire that will assess initial eligibility. Potentially eligible participants will be contacted by a member of the research team to explain more about the study and schedule a baseline appointment at which consent will be received.

### Eligibility and allocation

Inclusion and exclusion criteria are summarised in the box 1.

Box 1 **Participant eligibility criteria**

**Inclusion criteria**

- Children must:
  - be aged between 6 months and less than 12 years of age
  - have eczema diagnosed by an appropriately qualified healthcare professional (registered doctor, nurse or health visitor)
  - mild eczema or worse (patient-orientated eczema measure score >2 within previous 28 days)
- The person giving consent must:
  - have parental responsibility for the participant
  - be willing to use the randomly allocated emollient type as the only leave-on emollient for 16 weeks.

**Exclusion criteria**

- Child:
  - known sensitivity to study emollients or their constituents
  - participating in another research study currently or in the last 4 months
  - any other known adverse medical or social circumstance that would make invitation to the study inappropriate (as determined by GP surgery)
- The person giving consent:
  - unable to give informed consent
  - insufficient written English to complete outcome measures

Participants will be randomised in a 1:1:1:1 ratio to the four groups, stratified by centre and minimised by baseline patient-orientated eczema measure (POEM — mild 3 to 7 vs moderate/severe 8+)^10^ and participant age (less than 2 years old vs 2 years and above) using a validated web-based randomisation system supplied by the Bristol Randomised Trials Collaboration. Allocation is secure, concealed and cannot be changed once made.

### Intervention

In the NHS, GP prescribing is restricted by local formularies which vary widely and change over time. Therefore, participants will be randomised to a type of emollient (lotion, cream, gel or ointment) rather than a specific named emollient. However, to reduce heterogeneity within each type of emollient, GPs will be asked to only prescribe emollients which share certain characteristics (table 1). Study emollients will therefore be distinct between types and similar within each type. It would be considered unethical to withhold an emollient from a participant, and so there is no ‘control’ group.

**Table 1:** Rules for exclusion/inclusion of different types of emollients

Type of emollient		Lotion	Cream	Gel	Ointment
Rules/group shared characteristics	Exclusion	Antimicrobials or urea [should be centered so crosses lotion, crea, gel and ointment columns]			
	Inclusion	Paraffin-based [should be centered so crosses lotion, crea, gel and ointment columns]			
		Glycerol containing only	No humectant or lanolin	Does not contain povidine	No additives
Example formulary emollients from each group*		Cetraben lotion, QV lotion and Diprobase lotion	Diprobase cream, Epimax cream, Aquamax cream, Zerobase cream and AproDerm cream	Doublebase gel, Isomol gel, Zerodouble gel, AproDerm gel and MyriBase gel	Diprobase ointment, Emulsifying ointment BP, White soft/Liquid paraffin 50/50 ointment, Paraffin White soft ointment and Paraffin Yellow soft ointment

*Membership will be monitored and may change over time, keeping within the inclusion and exclusion criteria for each group.

At the baseline visit, the researcher will give parents simple verbal advice and a one-page summary on emollient use. GPs will issue a prescription of the study emollient with directions to ‘Use twice daily and as required’ and make it available for repeat prescription. This is consistent with usual care, where clinician advice usually does not extend beyond what is written on the prescription, sometimes backed-up with an information leaflet. Parents will be contacted within 1 week of randomisation to ensure that they have collected and started using the study emollient. The amount of emollient used during the study will be determined by the family.

Parents will be asked to agree to use the study emollient as the only leave-on emollient for 16 weeks. However, if the family have problems with or dislike their study emollient, they can stop it and seek an alternative from their GP. In this instance, the GP/family will be encouraged to try another emollient of the same type.

Clinical management of eczema will otherwise be as usual, with participants free to continue using or change other treatments. Use of other emollients as soap substitutes for washing only is permissible and will not be classed as contamination.

### Outcomes

The primary outcome is POEM, measured weekly for 16 weeks. POEM is a patient-reported outcome that can be completed by proxy (carer report) and captures symptoms of importance to parents and patients over the previous week.^11^ It demonstrates good validity, repeatability and responsiveness to change.^12 13^ We have chosen repeated measures because eczema is a relapsing and remitting long-term condition and this approach captures effectiveness of treatments better than comparing outcomes at a single time point.

Secondary outcomes include:

- Eczema Area Severity Index (EASI).
- Use of study emollient/other eczema treatments.
- Parent-reported satisfaction with study emollient.
- Adverse events: localised reactions, slips and falls.
- Child and family-oriented quality of life measures: Atopic Dermatitis Quality of Life (ADQoL)^14^; Dermatitis Family Impact questionnaire (DFI)^15^ and Child Health Utility 9D (CHU-9D).^16 17^

A complete schedule of data collection can be found in table 2. We are following-up participants for 1 year because eczema is a relapsing-remitting condition where symptoms can be seasonal and there is paucity of long-term outcome data in relation to emollient use in children with eczema.

**Table 2:** Schedule of enrolment, interventions and assessments

	Study period																												Close-out
	Enrolment	Allocation	Post-allocation																V_1_	Participant questionnaires									
	S	V_0_	Participant questionnaires																										
Week		0	1	2	3	4	5	6	7	8	9	10	11	12	13	14	15	16	16	20	24	28	32	36	40	44	48	52	
Parent completed																													
Screening questionnaire	●																												
Opinion about emollients		●																											
POEM	●	●	●	●	●	●	●	●	●	●	●	●	●	●	●	●	●	●		●	●	●	●	●	●	●	●	●	
Eczema pain & bother		●				●				●				●				●											
Use of treatments for eczema		●	●	●	●	●	●	●	●	●	●	●	●	●	●	●	●	●		●	●	●	●	●	●	●	●	●	
Adverse events			●	●	●	●	●	●	●	●	●	●	●	●	●	●	●	●		●	●	●	●	●	●	●	●	●	
Consultations (non-EMR)						●				●				●				●		●	●	●	●	●	●	●	●	●	
Personal costs						●				●				●				●		●	●	●	●	●	●	●	●	●	
DFI		●																●										●	
ADQoL		●						●										●										●	
CHU-9D		●						●										●											
Satisfaction with emollient																		●											
Study experiences																												●	
Researcher administered																													
Demographics and history		●																											
UK diagnostic criteria for AD		●																											
EASI		●																	●										
EMR notes review																													●
**Nested qualitative study**																													
Audio-recording		○																											
Round one interviews				← ○ →																									
Round two interviews																			← ○ →										

● =all participants; ○= sample of participants. S: screening stage (responses to written invitation letters and people responding to opportunistic invites); V0 and V1: research face-to-face baseline & follow-up visits.

* Botherscore, itch intensity, parent global assessment.

AD, atopic dermatitis; ADQoL, Atopic Dermatitis Quality of Life; CHU-9D, Child Health Utility 9D;DFI, Dermatitis Family Impact; EASI, Eczema Area Severity Index;EMR, electronic medical record; POEM, patient-orientated eczema measure.

### Participant timeline, data collection methods and participant retention

Participants will take part in the trial for 52 weeks, with the primary outcome collected over the first 16 weeks (figure 1).

Baseline data will be collected by the researcher using paper case report forms. Parents will be given the option of completing follow-up questionnaires either online or on paper. Parents are asked to complete weekly surveys for the first 16 weeks and then every 4 weeks between 16 and 52 weeks. With consent, participants’ electronic medical records will be reviewed for data on prescriptions, consultations and referrals.

Parents will be sent regular newsletters and receive automatic emails or text reminders when their questionnaires are due. In recognition of their time and to encourage retention, parents will be offered £10 vouchers at the baseline and 16 weeks. We will also offer the child a small gift, for example, ‘bee’ toy, of about £5 in value.

### Masking

Table 3 summarises who is masked to treatment allocation. Procedures to maintain masking to allocation will be written and followed. Researcher masking will be assessed using the Bang blinding index.^18^ Because parents, participants and treating clinicians will know the treatment allocation, un-masking procedures are not required.

**Table 3:** Masking to treatment allocation

Individual(s)	Status
Participating children, their parents and any treating clinician	Unmasked: The allocated emollient is prescribed by the participant’s GP and issued by local pharmacy as in usual care.
Clinical trials unit (CTU) database staff, trial coordinators, trial administrator and qualitative researcher	Unmasked: CTU staff will maintain the randomisation database. The trial coordinator/administrator will randomise participants and be the initial point of contact for all enquiries relating to issues with the emollients.
Qualitative team (Drs Sutton, Heawood and Banks)	Unmasked: Participants will be sampled based on emollient allocation/use and during the interviews the qualitative researcher will specifically ask about the different emollient types.
Junior statistician (Ms Sanderson)	Unmasked: The junior statistician was initially masked knowing only an anonymised code for the different treatment groups. After approval of the statistical analysis plan, she was unmasked to permit preparation and discussion of unmasked data with the data monitoring committee.
Trial manager and chief investigator	Masked: The trial manager was masked prior to the writing of the statistical analysis plan but is unmasked on an individual participant basis, when required to undertake randomisations and deal with potential serious adverse events. The chief investigator will only be unmasked in the event of a serious adverse event.
Other trial management group members: Dr MacNeill (senior statistician), Dr Santer & Professor Thomas (PIs), Ms Barrett (pharmacist), Dr Lane & Dr Taylor (CTU), Professors Hay & Williams (senior researchers), Ms Kirsty Garfield (health economist), Dr Baxter (knowledge mobilisation), Mrs Roberts (PPI)	Masked: Procedures will be put in place to maintain masking both within and outside of project meetings.
[INstead of reseacrcjers in column to left, please put Clinical Study Officers] Masked: Masking of researchers undertaking baseline and 16 week visits will be monitored by means of self-report.

GP, general practitioner; PIs, principal investigators; PPI, patient and public involvement.

### Sample size

As we have four groups, we powered our sample size calculation to detect a clinically meaningful differences in six pairwise comparisons subsequent to a global test. We estimate that 416 participants (104 in each group) are required to detect a difference of 3.0 in POEM scores^12 19 20^ between any two groups with 90% power and a significance level of 0.05 (after adjustment for multiple pairwise comparisons). We assumed a SD of 5.5 (SD of 4.89 observed in feasibility trial^21^ to allow for greater variability in the data or smaller differences to be detected. To allow for 20% loss to follow-up, we propose recruiting 520 patients in total.

### Data management

Personal data of participants’ and their parents will be treated as strictly confidential and entered onto a secure administrative database stored on the University of Bristol server. Anonymised trial data will be collected and managed using the study’s REDCap database.^22^ This system will also be used to administer online questionnaires for those who choose for online rather than paper questionnaires. The system incorporates data entry and validation rules to reduce data entry errors and management functions to facilitate auditing and data quality assurance.

### Statistical methods

The analysis and presentation of the trial data will be in accordance with Consolidated Standards of Reporting Trials guidelines.^23 24^ A full statistical analysis plan has been developed and approved by the independent statistician on the study’s trial steering committee ahead of analysis of post-randomisation data and will be made available via the study website.

Baseline characteristics of patients will be compared between the four arms by reporting summary statistics. Characteristics will be reported as means and SD, medians and IQRs or frequencies and proportions depending on the nature of the data and its distribution. If baseline characteristics of any two treatment groups differ by more than 10% or 0.5 SD then the effect of this variable on the primary outcome will be investigated in a sensitivity analysis.

Primary statistical analyses between the randomised groups will be conducted on an intention-to-treat basis. For the primary outcome we will use linear mixed models (weekly observations, level 1; nested within participants, level 2) to explore whether there are differences in mean POEM scores between treatment groups after adjusting for baseline scores and all stratification and minimisation variables used in the randomisation. Pairwise comparisons will be conducted to identify which intervention groups differed. To account for multiple testing, we will use a modified alpha of 0.0083 (0.05/6 pairwise comparisons equivalent).

Secondary outcomes will be analysed according to the data type and frequency of recording. Continuous outcomes measured at multiple time points will be analysed similarly to the primary outcome as described above. Continuous outcomes measured once after randomisation — such as EASI score — will be analysed using linear regression adjusting for baseline values where available. We will consider alternative methods should assumptions not be met.

To assess adherence to the allocated medication, for each participant, we will count the number of days of self-reported use of the allocated type of emollient and express that as a proportion of the number of days for which non-missing emollient use data are available. Contamination will be assessed by calculating the proportion of days (among days where non-missing emollient use data are available) where a non-allocated emollient type was used. We are unable to prespecify what constitutes ‘substantial contamination’, which may inform further sensitivity analyses.

Other proposed sensitivity analyses include an exploration of patterns of missing data and we will consider possible mechanisms for this. Based on these and observed data, appropriate methods for imputing missing data will be considered in sensitivity analyses. Also, should there be evidence of imbalance between treatment groups on important baseline characteristics we will conduct a regression analysis of the primary outcome adjusting additionally for these variables.

Descriptive analysis of safety endpoints will be presented according to randomised group. Prespecified subgroup analyses will investigate whether treatment effectiveness is modified by the following factors measured at randomisation: parent expectation, age of child at randomisation, disease severity and eczema diagnosis. These subgroup analyses will involve incorporating interaction terms with treatment allocation to test the null hypothesis of no variation in treatment effect across subgroups. These tests are likely to be underpowered, however, therefore emphasis will be placed on the point estimates and confidence intervals generated.

### Nested qualitative study

The aims of the qualitative study are first, to support and optimise participant recruitment and retention; and second, to complement, explain and aid understanding of the quantitative findings

#### Baseline appointment recordings

To meet the first aim, a sample of baseline appointments (at least one per recruiting researcher) will be audio-recorded and reviewed by a qualitative researcher. Using a structured template, the interaction will be reviewed to ensure key information is relayed and parent understanding checked. Recommendations will be feedback individually to the relevant researcher and collectively (anonymised) to other recruiting researchers and the trial management group. Prior to the start of the baseline appointment, parents will be asked to give verbal consent for the recording, with written consent obtained at the end of the appointment.

#### Interviews with parents and trial participants

To meet the second aim, we will interview parents and, at their discretion, the participating children themselves, at 4 weeks and 16 weeks after randomisation. The design is cross-sectional, with different families interviewed at each time point. However, where particularly interesting issues emerge, we may speak to a family at both time points. Parents will indicate on the trial consent form whether they are willing to be approached for these.

The 4 week interviews will focus on the initial use and acceptability of the assigned emollient. We will conduct up to five interviews in each trial group (total ~20), purposively sampling by: recruitment centre, age of child, eczema severity and allocated type of emollient. We will include those who have stopped using the allocated treatment or switched emollient.

The 16 week interviews will focus on the overall experience of using the assigned emollient, perceived effectiveness, planned future use of emollients and experience of taking part in the trial. The sampling criteria will be the same as for the 4 week interviews, with the additional criterion of intentions regarding future emollient use. We expect to achieve data saturation by conducting up to 10 interviews in each trial group (total ~40).

Interviews are expected to last between 30 to 60 min. Topic guides (including subtopic guide for children) will be used but with flexibility to allow unanticipated issues to emerge and be further explored in later interviews. Interviews will be captured using an encrypted digital voice recorder, transcribed and anonymised to protect confidentiality.

The interview data will be analysed thematically, using a combination of deductive and inductive coding^25^ and adapted techniques of constant comparison.^26^ Analysis will be led by the qualitative researcher, with input from the qualitative co-applicants and trial management group. Data management and coding will be aided by use of NVivo software. Data will be compared within and across trial group, with attention to converging and diverging perspectives. The themes will be written up as interpretive summaries with illustrative verbatim quotes that represent the range of expressed views.

### Monitoring, safety and audit

As the randomised treatments within this study do not differ from common usual clinical practice, risk-based monitoring will be implemented in line with a risk-assessment. Data on adverse events will be collected by parent self-report. No interim analyses are planned.

An independent Data Monitoring Committee has been established and terms of reference have been drawn up and agreed. The committee will meet at least annually, and its role is to safeguard the interests of the trial’s participants, potential participants, investigators and sponsor; to assess the safety and efficacy of the trial’s interventions, and to monitor the trial’s overall conduct, and protect its validity and credibility.

The sponsor organisation is the University of Bristol. Adverse event reporting will be in accordance with local procedures.

The trial may be prematurely discontinued due to lack of recruitment or by the sponsor, chief investigator, regulatory authority or funder based on new safety information or for other reasons given by the trial steering committee or data monitoring committee, regulatory authority or ethics committee concerned.

### Public and patient involvement

In 2013, the James Lind Alliance published the eczema research priorities for patients and healthcare professionals and ‘Which emollients are the most effective and safe in treating eczema?’ emerged as one of the highest ranked uncertainties.^27^

Co-author AR is mother of children with eczema and a member of Nottingham Support Group for Carers of Children with Eczema. We have established a group of parents of children with eczema, who helped develop the study and want to support our ongoing work through meetings and email communication. A patient and public involvement (PPI) member sits on the trial steering committee. We will use the internet and social media to promote wider patient engagement.

PPI has helped us to frame the research question around, ‘Which emollient to prescribe first?’ for childhood eczema, acknowledging that individuals differ in their experiences of effectiveness and tolerability of different emollients. It has also gave us a clear steer that including a non-emollient group would be unacceptable to many families, favoured POEM as the primary outcome and highlighted how emollient use may be a ‘trade off’ between effectiveness and acceptability.

Ongoing PPI involvement has informed both qualitative and quantitative data collection and helps ensure that the study continues to focus on delivering clinically important outcomes that are meaningful to patients.^28^

## Ethics and dissemination

## Protocol amendments

Any amendments to the protocol will be reported accordingly to the regulatory bodies, with a copy of the current protocol (V.6.0 currently) available for download from the study website. Amendments to date are listed in online supplementary appendix 1.

10.1136/bmjopen-2019-033387.supp1 Supplementary data

### Consent and assent

Written consent for taking part in the trial will be received by a researcher from the parent or guardian of the participant at their baseline appointment. For children approximately 7 years and older, the option of providing assent will be offered alongside parental consent (see online supplementary appendix 2).

10.1136/bmjopen-2019-033387.supp2 Supplementary data

### Confidentiality and access to data

The database and randomisation system will protect patient information in line with the data protection legislation. Trial staff will ensure that participants’ anonymity is maintained through protective and secure handling and storage of patient information at the lead centre. The chief investigator will have access to and act as custodian of the full data set.

### Ancillary and post-trial care

After the 16 week primary outcome period, participants will be free to change their emollient if they wish. Conversely, they will be able to continue with their allocated emollient after they have completed follow-up.

### Dissemination and data sharing

A series of stakeholder meetings will raise study awareness among and share progress and findings with policymakers, voluntary groups, clinicians, patients, families. Study progress, outputs and a summary of findings will be made available via a study website and Twitter account; and summaries distributed to participating families and GP surgeries. Findings will be submitted for presentation at conferences and written up for publication in a peer-reviewed journal(s), which may include integration of the quantitative and qualitative findings. The International Committee of Medical Journal Editors has criteria for authorship will be observed and no professional writers will be employed.

No later than 3 years after the completion of the study, we will deposit a de-identified data set in an appropriate data archive.

## Discussion

Factors that may influence patient preference for different types of emollient include disease severity, body site, cosmetic acceptability of the product, season/climate and packaging.^29^ Cultural factors may also influence choice and use.^30^ NICE recommends patients try different emollients in the clinic before choosing.^5^ This approach is not practical in primary care, and even in specialist settings the range of emollients available to try can be arbitrary — restricted by local formularies and influenced of pharmaceutical companies. Therefore, most patients consulting in primary care are unaware of differences between emollients; and many primary care clinicians will be unable to advise on grounds other than consistency or simple unit cost.

Some emollients are decades old and it has not been in the interest of manufacturers to submit their products in a head-to-head comparison with others in a clinical trial. In BEE, we are independently evaluating in a pragmatic trial, using a validated patient-reported primary outcome, the effectiveness of the four types of emollients commonly prescribed for children with eczema. In accordance with the recommendations of Harmonising Outcome Measures in Eczema, POEM and EASI will be used to measure patient-reported symptoms and clinical signs, respectively.^31^

Participants are unmasked, so knowing which emollient they’re using may bias assessment of emollient effectiveness. However, we have chosen a patient-reported outcome as the primary outcome because symptoms of eczema are more important to families of than objective measures which are based on skin appearance.^11^ We will minimise the potential for performance bias by ensuring that at the point of consent parents are willing to use any of the four emollients for the first 16 weeks. We will also measure at baseline parent opinion regarding the four different study emollients, and in a subgroup analysis explore whether reported effectiveness is linked to high/low prior expectations of effectiveness. The collection of an objective measure of eczema severity (EASI) by a masked researcher as a secondary outcome allows us to examine outcomes in relation to signs of eczema. Subject to additional funding, we plan to undertake a full economical evaluation to determine the cost-effectiveness of the four emollient types.

Recruitment started in January 2018 and follow-up of the last participant is scheduled by February 2021. Findings from the BEE study, comparing the clinical effectiveness and acceptability of commonly used different emollients, will provide evidence on which clinicians and carers/patients can decide which emollient to try first. Our aim is not to reduce choice, but to reduce uncertainty and the consequences of ‘trial and error’ prescribing. Smarter prescribing will help prescribers and carers gain ‘control’ over the eczema more quickly, reduce frustration and inconvenience for families and potentially produce cost savings to the NHS through cost-effective prescribing and fewer repeat consultations to change emollients.

## Supplementary Material

Reviewer comments

Author's manuscript
