# Supplementary material for: Best emollients for eczema (BEE) – comparing four types of emollients in children with eczema: protocol for randomised trial and nested qualitative study
Source: BMJ Open. 2019 Nov 6;9(11):e033387. doi: 10.1136/bmjopen-2019-033387 (PMC6858146; doi:10.1136/bmjopen-2019-033387)
Supplement: Supplementary data [file bmjopen-2019-033387supp001.pdf]

Appendix 1: Administrative information

Title

The Best Emollient for Eczema (BEE) trial: a randomised trial comparing the effectiveness of four types of commonly prescribed emollients for children with eczema

Trial registration number

ISRCTN: ISRCTN84540529 (Date registered: 05/06/2017)

World Health Organization Trial Registration Data Set

Data category	Information
Primary registry and trial identifying number	ISRCTN84540529
Date of registration in primary registry	05.06.2017
Secondary identifying numbers	EudraCT: 2017-000688-34
Source(s) of monetary or material support	NIHR Health Technology Assessment (HTA) 15/130/07
Primary sponsor	University of Bristol
Secondary sponsor(s)	Not applicable
Contact for public queries	bee-study@bristol.ac.uk, 0117 928 7351
Contact for scientific queries	Dr Matthew Ridd FRCGP PhD, m.ridd@bristol.ac.uk, 0117 331 4557
Public title	Best Emollients for Eczema (BEE) study
Scientific title	The Best Emollient for Eczema (BEE) trial: a randomised trial comparing the effectiveness of four types of commonly prescribed emollients for children with eczema
Countries of recruitment	England
Health condition(s) or problem(s) studied	Childhood eczema
Intervention(s)	Lotion, cream, gel or ointment as the only leave-on emollient for 16 weeks with directions to apply twice daily and as required.
Key inclusion and exclusion criteria	CHILD<br>Inclusion criteria:<br>1. Aged between 6 months and less than 12 years of age<br>2. Have eczema diagnosed by an appropriately qualified healthcare professional (registered doctor, nurse or health visitor)<br>3. Have mild eczema or worse (POEM score>2)<br><br>Exclusion criteria:<br>1. Known sensitivity to study emollients or their constituents<br>2. Participating in another research study currently or in the last four months<br>3. Any other known adverse medical or social circumstance that would

	make invitation to the study inappropriate (as determined by GP practice staff)<br><br>PERSON GIVING CONSENT:<br>Inclusion criteria:<br>1. Have parental responsibility for the participant<br>2. Willing to use the randomly allocated emollient as the only leave-on emollient for 16 weeks.<br><br>Exclusion criteria:<br>1. Unable to give informed consent<br>2. Insufficient written English to complete outcome measures.
Study type	Intervention
Date of first enrolment	19 January 2018
Target sample size	520
Recruitment status	Recruiting
Primary outcome(s)	Eczema symptoms, measured using POEM over 16 weeks
Key secondary outcomes	Eczema symptoms, measured using POEM over 52 weeks; eczema signs, measured using EASI; eczema 'bother' score; itch intensity score; parent global assessment of eczema; other possible symptoms of food allergy; UK diagnostic criteria for atopic dermatitis; main carer anxiety, measured using GAD-7; diet of child and/or mother if child being breastfed by her; adverse events; child and family quality of life, measured using ADQoL, CHU-9D and IDQoL; satisfaction with trial processes, procedures and paperwork; health services utilisation; out-of-pocket expenses/time off work.

Protocol version and history of amendments

The current version of the protocol is 6.0 (10.06.19). Previous protocols and amendments are as below:

Version		Notes
**Number**	**Date**	
1.0	21.03.2017	Submitted for approval (March 2017) and approval received from REC, MHRA and HRA.
2.0	27.06.2017	Title page: ISRCTN, NHS REC, and NIHR portfolio numbers added; 10.3: Clarification of eligibility confirmation; 10.6: "Blinding to treatment allocation" table amended to reflect changes in research team/processes to minimize un-blinding of TMG members, in accordance with TS/DM-C recommendation; 12.3: Clarification that first set of interviews will be with participants during their first four weeks in the study, not during the first four weeks of the life of the trial itself; 19.2: clarity to TS/DM-C composition/roles; 14.3: clarification about who makes decisions regarding causality of adverse events/reactions.
3.0	03.08.2017	Clarification that screening POEM must be within 28 days of recruitment. Removal of signature page to separate document.
4.0	03.11.17	Amendment to the intervention, from 4 specific emollients, to type of emollient. Correction of minor typos. Clarification of Safety reporting

Ridd MJ, *et al. BMJ Open* 2019; 9:e033387. doi: 10.1136/bmjopen-2019-033387

		section. Update to "Timetable and milestones" to reflect delayed start to internal pilot.
5.0	01.08.18	Change "Bristol CCG" to "Bristol, North Somerset and South Gloucestershire CCG" to reflect merger/name change (page 6); changes to blinding arrangements (**Error! Reference source not found.**) and removal of reference to "Ms Jameson", former CAPC PPI&E coordinator who was never a TMG member and has subsequently left (page 27/28); update to section 19.2 (Oversight committees) to describe separate TSC and DMC created at request of funder after approval of protocol 4.0; other minor grammatical/style changes/corrections.
6.0	10.06.19	Updated references to timelines throughout to reflect 38-months recruitment and follow-up / 50-month total study duration. Insertion of paragraph on participant communication (section 10.8, Participant stipends and communication). Replace Avon Primary Care Research Collaboration logo with BNSSG CCG logo. Replace any reference to blind, blinded or blinding with masked or masking. Extra information for parents of study participants in order to bring study in-line with the EU General Data Protection Regulations 2018. Minor changes to titles/postal addresses.

Funding

NIHR Health Technology Assessment (HTA) 15/130/07

Contributorship

See main manuscript

Sponsor contact information

Trial sponsor: University of Bristol
Sponsor's reference: 2738
Contact name: Dr Rachel Davies
Address: Research Enterprise Development, One Cathedral Square, Bristol BS1 5DD
Email: research-governance@bristol.ac.uk
Telephone: 0117 428 4011

Role of study sponsor and funder

The funder and sponsor had no role in the design of this study and will not have any role during its execution, analyses, interpretation of the data, or decision to submit results.

Committees

The study is hosted by BNSSG CCG, and will be delivered by the University of Bristol, in collaboration with partners at University of Nottingham, University of Southampton. The Universities of Nottingham and Southampton will be recruiting centres, with Professor Kim Thomas and Dr Miriam Santer as the Principal Investigators, respectively.

The Trial Management Group comprises all investigators, the trial manager, research and administrative staff, the trials unit and patient/public representative. Members will contribute to the trial in the following ways: trial design and methods; participant recruitment and trial conduct; trial management; trial logistics and cost management; economic evaluation; qualitative study statistical data analysis; and publication. The Trial Management Group will meet on a regular basis to

oversee the management of the trial. Meetings will be face-to-face with teleconference facilities for TMG members who are unable to be present.

This study was designed and is being delivered in collaboration with the Bristol Randomised Trials Collaboration, a UKCRC registered clinical trials unit which, as part of the Bristol Trials Centre, is in receipt of National Institute for Health Research Clinical Trials Unit support funding.

Because this is a low-risk trial, the funder originally agreed that the roles of both guiding the TMG and monitoring trial data will be undertaken by a single joint committee, the Trial Steering/Data Monitoring Committee. However, because of changes implemented in version 4.0 of the protocol, the funder requested that separate Trial Steering and Data Monitoring Committees be established.

The Trial Steering Committee will provide overall supervision of the trial on behalf of the funder. Terms of reference have been drawn up and agreed with members, which comprises four independent members: a chairperson, an academic, a biostatistician and a patient representative (parent of child with eczema). There is one additional non-independent member who is a qualitative researcher. Non-independent members will not have any voting rights. The Trial Steering Committee will meet at least four times over the course of the study, including one which will coincide with the end of the internal pilot and a final meeting, when analysis is almost complete and the final report is being prepared.

The Data Monitoring Committee will safeguard the interests of the trial's participants, potential participants, investigators and sponsor; to assess the safety and efficacy of the trial's interventions, and to monitor the trial's overall conduct, and protect its validity and credibility. Terms of reference have been drawn up and agreed with members, which comprises three independent members: a chairperson, a biostatistician and GP with specialist interest in dermatology. The Data Monitoring Committee will meet at least annually: only committee members and the junior statistician should be present in closed sessions; open sessions will be attended by those at the closed session, plus the CI and possibly representatives of the sponsor or funder, and a trial unit representative.

Ridd MJ, *et al. BMJ Open* 2019; 9:e033387. doi: 10.1136/bmjopen-2019-033387
