# Supplementary material for: Best emollients for eczema (BEE) – comparing four types of emollients in children with eczema: protocol for randomised trial and nested qualitative study
Source: BMJ Open. 2019 Nov 6;9(11):e033387. doi: 10.1136/bmjopen-2019-033387 (PMC6858146; doi:10.1136/bmjopen-2019-033387)
Supplement: Supplementary data [file bmjopen-2019-033387supp002.pdf]

University of BRISTOL · UNIVERSITY OF Southampton · The University of Nottingham UNITED KINGDOM · CHINA · MALAYSIA · Bristol Randomised Trials Collaboration

BEE Best Emollient for Eczema

Best Emollient for Eczema (BEE) Study

Parent/Carer Consent Form

Initial box

1. I confirm that I have read and understand the Participant Information Sheet dated 03.11.2017 (version 3.0) for the above study. I have had the opportunity to consider the information, ask questions and have had these questions answered satisfactorily. ☐
2. I understand that participation is voluntary and that we are free to withdraw at any time without giving any reason, without my child's medical care or legal rights being affected. ☐
3. I understand that after the study ends, the data collected will be made "open data". I understand that this means the anonymised data will be publicly available and may be used for purposes not related to this study, and it will not be possible to identify me from these data. ☐
4. I understand that relevant sections of my child's medical notes and all information collected for this research may be reviewed by the study team, from regulatory authorities or from the NHS Trust for the purpose of ensuring that the research is conducted appropriately. I give permission for these individuals to access my child's records as appropriate. ☐
5. I give permission for researchers working on this study to have access to my child's medical records for the purposes of collecting information relevant to the aims of this study. ☐
6. I give consent for the data collected in this trial to be used in future ethically approved studies on the understanding that all information will continue to be kept securely and remain confidential. ☐
7. I give consent to be contacted by a member of the research team with a view to being interviewed about my experiences of emollients and taking part in BEE. I understand that I will be given more information first, I can decide later about taking part in this, I will be asked to give further consent for taking part in interviews and that I may not be contacted at all. ☐
8. *For those asked to take part in audio-recording of recruitment visit only:* I agree to have my recruitment visit audio-recorded, including anything my child may say. I agree to data from my audio-recorded interview being transferred to and retained by the Universities of Bristol, Southampton and Nottingham for training, teaching and research purposes, now and in the future. ☐
9. I agree for my child to take part in the above-named study. ☐

Name of Participant (Child)	Participant ID	
Name of Parent/Guardian	Signature	Date
Name of person receiving consent	Signature	Date

Funded by NHS National Institute for Health Research

The research was funded by the NIHR Health Technology Assessment (HTA) Programme. The views expressed are those of the author(s) and not necessarily those of the NHS, the NIHR or the Department of Health.

Parent Consent Form, Version 3.0, 03.11.17
IRAS 214900

Best Emollient for Eczema (BEE) Study

Assent Form for Children

(Assent means you are agreeing to join this study)

Please circle Yes or No for each question

1.	I have read the leaflet that explains about the BEE study.	Yes	No
2.	I have been able to ask questions about it.	Yes	No
3.	I understand what the study is all about.	Yes	No
4.	I understand that I do not have to take part if I do not want to.	Yes	No
5.	I can change my mind and I do not have to say why.	Yes	No
6.	I agree to take part in the study.	Yes	No

Name of Participant (Child) Signature Participant ID

Name of person receiving assent Signature Date

Child

Funded by NHS National Institute for Health Research

The research was funded by the NIHR Health Technology Assessment (HTA) Programme. The views expressed are those of the author(s) and not necessarily those of the NHS, the NIHR or the Department of Health.

Assent Form, Version 1.0, 21.03.17; IRAS 214900
