# Supplementary material for: Best emollients for eczema (BEE) – comparing four types of emollients in children with eczema: protocol for randomised trial and nested qualitative study
Source: BMJ Open. 2019 Nov 6;9(11):e033387. doi: 10.1136/bmjopen-2019-033387 (PMC6858146; doi:10.1136/bmjopen-2019-033387)
Supplement: Author's manuscript [file bmjopen-2019-033387.draft_revisions.pdf]

BMJ Open is committed to open peer review. As part of this commitment we make the peer review history of every article we publish publicly available.

When an article is published we post the peer reviewers' comments and the authors' responses online. We also post the versions of the paper that were used during peer review. These are the versions that the peer review comments apply to.

The versions of the paper that follow are the versions that were submitted during the peer review process. They are not the versions of record or the final published versions. They should not be cited or distributed as the published version of this manuscript.

BMJ Open is an open access journal and the full, final, typeset and author-corrected version of record of the manuscript is available on our site with no access controls, subscription charges or pay-per-view fees (http://bmjopen.bmj.com).

If you have any questions on BMJ Open's open peer review process please email info.bmjopen@bmj.com

BMJ Open

Best Emollients for Eczema (BEE) – comparing four types of emollients in children with eczema: protocol for randomised trial and nested qualitative study

Journal:	*BMJ Open*
Manuscript ID	bmjopen-2019-033387
Article Type:	Protocol
Date Submitted by the Author:	02-Aug-2019
Complete List of Authors:	Ridd, Matthew; University of Bristol, Population Health Sciences; University of Bristol<br>Wells, Sian; University of Bristol, School of Social & Community Medicine<br>Edwards, Louisa; Simon Fraser University, Faculty of Health Sciences; Centre for Clinical Epidemiology and Evaluation (C2E2),<br>Santer, Miriam; University of Southampton, Primary Care & Population Sciences<br>MacNeill , Stephanie; University of Bristol, Bristol Randomised Trials Collaboration, Bristol Trials Centre<br>Sanderson, Emily; University of Bristol, Bristol Randomised Trials Collaboration, Bristol Trials Centre<br>Sutton, Eileen; University of Bristol, Population Health Sciences; University of Bristol<br>Shaw, Ali; University of Bristol, School of Social and Community Medicine<br>Banks, Jonathan; University of Bristol, Social & Community Medicine<br>Garfield, Kirsty; University of Bristol, Bristol Randomised Trials Collaboration<br>Roberts, Amanda; University of Nottingham, Centre of Evidence-Based Dermatology<br>Barrett, Tiffany; University Hospitals Bristol NHS Foundation Trust, South West Medicines Information & Training<br>Baxter, Helen; University of Bristol, Bristol Medical School: Population Health Sciences; University of Bristol<br>Taylor, Jodi; University of Bristol, School of Clinical Sciences<br>Lane, Athene; University of Bristol, School of Social and Community Medicine<br>Hay, Alastair; University of Bristol, Centre for Academic Primary Care<br>Williams, HC; University of Nottingham,<br>Thomas, Kim; University of Nottingham, Dermatology
Keywords:	Eczema < DERMATOLOGY, Paediatric dermatology < DERMATOLOGY, THERAPEUTICS

SCHOLARONE™
Manuscripts

Title page

Title

Best Emollients for Eczema (BEE) – comparing four types of emollients in children with eczema: protocol for randomised trial and nested qualitative study

Authors

Matthew J Ridd,[1] Sian Wells,[1] Louisa Edwards,[2] Miriam Santer,[3] Stephanie J MacNeill,[4] Emily Sanderson,[4] Eileen Sutton,[1] Alison R G Shaw,[1] Jonathon P Banks,[6] Kirsty Garfield,[4] Amanda Roberts,[7] Tiffany J Barrett,[8] Helen Baxter,[1] Jodi Taylor,[4] J Athene Lane,[4] Alastair D Hay,[1] Hywel C Williams,[5] Kim Thomas[5]

[1] Centre for Academic Primary Care, Bristol Medical School, University of Bristol, UK.

[2] Faculty of Health Sciences, Simon Fraser University, Burnaby, Canada.

[3] Primary Care and Population Sciences, University of Southampton, Southampton, UK

[4] Bristol Randomised Trials Collaboration, Bristol Trials Centre, University of Bristol, Bristol, UK.

[5] Centre of Evidence Based Dermatology, University of Nottingham, Nottingham, UK

[6] National Institute for Health Research Collaborations for Leadership in Applied Health Research and Care West (NIHR CLAHRC West), University Hospitals Bristol NHS Foundation Trust, Bristol, UK

[7] Nottingham Support Group for Carers of Children with Eczema, Nottingham, UK.

[8] South West Medicines Information & Training, University Hospitals Bristol NHS Foundation Trust, Bristol, UK

Corresponding author

Matthew Ridd, GP and Reader in Primary Healthcare

Postal address: Centre for Academic Primary Care, Population Health Sciences, Bristol Medical School, University of Bristol, Canynge Hall, 39 Whatley Road, Bristol BS8 2PS

Email: m.ridd@bristol.ac.uk

Telephone: 0117 331 4557

Word count

Excluding title page, abstract, references, figures and tables: 3810

Keywords

Atopic eczema/dermatitis, food allergy, feasibility RCT

For peer review only - http://bmjopen.bmj.com/site/about/guidelines.xhtml

For peer review only

For peer review only - http://bmjopen.bmj.com/site/about/guidelines.xhtml

ABSTRACT

Background: Atopic dermatitis/eczema affects around 20% of children and is characterised by inflamed, dry, itchy skin. Guidelines recommend "leave on" emollients that are applied directly to the skin to add or trap moisture and used regularly, they can soothe, enhance the skin barrier, and may prevent disease "flares". However, the suitability of the many different emollients varies between people and there is little evidence to help prescribers and parents and carers decide which type to try first.

Methods and analysis: Design: pragmatic, multi-centre, individually randomised, parallel group superiority trial of four types of emollient (lotions, creams, gel or ointments). Setting: GP surgeries at three sites in England. Participants: children aged over 6 months and less than 12 years with mild to severe eczema and no known sensitivity to study emollients. Interventions: study-approved lotion, cream, gel or ointment as the only leave-on emollient for 16 weeks, with directions to apply twice daily and as required. Other treatments, such as topical corticosteroids, used as standard care. Follow-up: 52 weeks. Primary outcome: validated parent-reported eczema symptoms (POEM) measured weekly for 16 weeks. Secondary outcomes: eczema signs (EASI) by masked researcher, treatment use, parent satisfaction, adverse events, child and family quality of life (ADQoL, CHU-9D and DFI). Sample size: 520 participants (130 per group). Analysis: intention-to-treat using linear mixed models for repeated measures. Nested qualitative study: audio-recording of sample of baseline appointments and up to 60 interviews with participants at four and 16 weeks, interviews to be transcribed and analysed thematically.

Ethics and dissemination: Ethics approval granted by the NHS REC (South West - Central Bristol Research Ethics Committee 17/SW/0089). Findings will be presented at conferences, published in open-access peer-reviewed journals and the study website; and summaries shared with key stakeholders.

Trial registration: ISRCTN: ISRCTN84540529 (Date registered: 05/06/2017)

Word count: 286/300

Strength and limitations of this study

- First, adequately powered head-to-head pragmatic trial of the four main types of emollient prescribed for the treatment of eczema in children, recruited from primary care, with long-term follow-up.
- The primary core outcome is a validated patient-reported measure (POEM) that captures symptoms of eczema that matter to patients, and weekly measures over the 16-weeks mean that all participants who complete at least one POEM post-baseline will be included in the analysis.
- Researchers undertaking assessments of eczema signs (secondary outcome) are masked to allocation and use validated core outcome (EASI).
- Parents and their clinicians are unmasked and therefore their assessment of both the effectiveness and acceptability of the study emollient may be biased.
- Study emollients of each type are similar, increasing generalisability of the findings, but because they are not identical subtle differences both within and between-types may not be identified.
- The findings will reduce "trial-and-error" prescribing of initial choice of emollient but should not be used to restrict emollient options.

For peer review only - http://bmjopen.bmj.com/site/about/guidelines.xhtml

Main text

INTRODUCTION

Background and rationale

Eczema affects around 20% of children.[1] It is characterised by dry and inflamed itchy skin, and it can have a significant impact on the quality of life for both the child and their family.[2] In accordance with the recommended nomenclature of the World Allergy Organisation, we use the label "eczema" to refer to the clinical phenotype of atopic eczema/dermatitis.[3]

The majority of children with eczema have disease of mild or moderate severity and are diagnosed and managed exclusively in primary care.[4] Children are commonly prescribed a moisturiser (emollient) and topical corticosteroid/topical calcineurin inhibitor to use alongside to treat or prevent "flares".[5] By direct application to the skin, emollients improve skin hydration and reduce symptoms such as stinging or itching, but they can also act as a barrier to potential irritants. Mild anti-inflammatory properties may reduce reliance on topical corticosteroids/calcineurin inhibitors.[6] Many directly applied or "leave-on" emollients can also be used as soap substitutes.

However, there are many different emollients available and little evidence that any one emollient is better than another as a leave on treatment. The main formulations are lotions, creams, gels and ointments, which vary in their consistency from "light" to "heavy". This mainly reflects differences in their oil (lipid) to water ratios. Some products also contain humectants which help retain moisture, but emollients containing urea or antimicrobial compounds tend to be reserved for more severe disease.

The absence of evidence regarding the comparative clinical and cost-effectiveness of different products is reflected in emollient formularies. Clinician prescribing in the NHS is guided by locally produced and maintained formularies, which recommend which items should be prescribed in that area. In 2018, across England and Wales there were over 100 different emollient formularies which made widely varying recommendations about 109 different emollients.[7] The current situation where healthcare professionals recommend different emollients and carers find an effective emollient through a process of "trial and error" is detrimental to both families and the NHS.[8 9]

In 2007, NICE recommended research to identify "the most effective and cost-effective combinations of emollient products to use for the treatment of childhood atopic eczema".[5] A recently published Cochrane review identified 77 trials, comprising 6603 participants, evaluating the effectiveness of emollients.[6] The authors were unable to conclude whether some of the moisturisers, or their ingredients, are better than others, and recommended head to head comparisons in clinical trials.

Aim and objectives

The aim of the study is to compare the effectiveness and acceptability of four types of emollient (lotion, cream, gel and ointment) most commonly used to treat eczema.

The objectives are to compare the four different emollient types, over the medium (16 weeks) and long-term (52 weeks), with respect to:

- Parent-reported eczema symptoms
- Researcher assessment of eczema signs

For peer review only - http://bmjopen.bmj.com/site/about/guidelines.xhtml

- Quality of life for the child
- Impact of eczema on the family
- Adverse effects
- Acceptability of and parent satisfaction with study emollient
- Frequency and quantity of study emollient and other emollient use
- Use of other eczema treatments (including topical corticosteroid and calcineurin inhibitor)
- Number of well-controlled weeks

Trial design

BEE is a pragmatic, multi-centre, individually randomised, parallel group superiority trial of four types of emollient in children with eczema, with nested qualitative study.

It is a type A Clinical Trial of an Investigational Medicinal Product (CTIMP) trial, which is low risk because the use of the medicinal product is not higher than the risk of standard medical care.

METHODS AND ANALYSIS

Study setting

Primary care (GP surgeries) in and around Bristol, Southampton and Nottingham

Recruitment

The stages of participant recruitment are shown in Figure 1.

We will identify children aged between 6 months and less than 12 years with eczema via an electronic medical records search. A GP or a delegated member of the practice team will screen the search results for inclusion/exclusion criteria. Parents and carers (hereafter parents) of potentially eligible children will be posted an invitation. In addition, GPs can recruit participants opportunistically.

Interested parents will complete a brief screening questionnaire that will initially assess eligibility. Potentially eligible participants will be contacted by a member of the research team to explain more about the study and schedule a baseline appointment at which consent will be received.

Eligibility and allocation

Inclusion and exclusion criteria are summarised in the Box.

Participants will be randomised in a 1:1:1:1 ratio to the four groups, stratified by centre and minimised by baseline Patient Orientated Eczema Measure (POEM – mild 3-7, versus moderate/severe 8+)[10] and participant age (less than 2 years old versus 2 years and above) using a validated web-based randomisation system supplied by the Bristol Randomised Trials Collaboration. Allocation is secure, concealed and cannot be changed once made.

Intervention

In the NHS, GP prescribing is restricted by local formularies which vary widely and change over time. Therefore, participants will be randomised to a type of emollient (lotion, cream, gel or ointment) rather than a specific named emollient. However, to reduce heterogeneity within each type of emollient, GPs will be asked to only prescribe emollients which share certain characteristics (Table 1). Study emollients will therefore be distinct between types and similar within each type. It would

For peer review only - http://bmjopen.bmj.com/site/about/guidelines.xhtml

be considered unethical to withhold an emollient from a participant, and so there is no "control" group.

At the baseline visit, the researcher will give parents simple verbal advice and a one-page summary on emollient use. GPs will issue a prescription of the study emollient with directions to "Use twice daily and as required" and make it available for repeat prescription. This is consistent with usual care, where clinician advice usually does not extend beyond what is written on the prescription, sometimes backed-up with an information leaflet. Parents will be contacted within one week of randomisation to ensure that they have collected and started using the study emollient. The amount of emollient used during the study will be determined by the family.

Parents will be asked to agree to use the study emollient as the only leave-on emollient for 16 weeks. However, if the family have problems with or dislike their study emollient, they can stop it and seek an alternative from their GP. In this instance, the GP/family will be encouraged to try another emollient of the same type.

Clinical management of eczema will otherwise be as usual, with participants free to continue using or change other treatments. Use of other emollients as soap substitutes for washing only is permissible and will not be classed as contamination.

Outcomes

The primary outcome is the POEM, measured weekly for 16 weeks. POEM is a patient-reported outcome that can be completed by proxy (carer report) and captures symptoms of importance to parents and patients over the previous week.[11] It demonstrates good validity, repeatability and responsiveness to change.[12 13] We have chosen repeated measures because eczema is a relapsing and remitting long-term condition and this approach captures effectiveness of treatments better than comparing outcomes at a single time point.

Secondary outcomes include:

- Eczema Area Severity Index (EASI)
- Use of study emollient/other eczema treatments
- Parent-reported satisfaction with study emollient
- Adverse events: localised reactions, slips and falls
- Child and family-oriented quality of life measures: Atopic Dermatitis Quality of Life (ADQoL);[14] Dermatitis Family Impact questionnaire (DFI)[15] and Child Health Utility 9D (CHU-9D),[16 17]

A complete schedule of data collection can be found in Table 2. We are following-up participants for one year because eczema is a relapsing-remitting condition where symptoms can be seasonal and there is paucity of long-term outcome data in relation to emollient use in children with eczema.

Participant timeline, data collection methods and participant retention

Participants will take part in the trial for 52 weeks, with the primary outcome collected over the first 16 weeks (Figure 1).

Baseline data will be collected by the researcher using paper case report forms (CRFs). Parents will be given the option of completing follow-up questionnaires either online or on paper. Parents are asked to complete weekly surveys for the first 16 weeks and then every 4 weeks between 16 and 52

For peer review only - http://bmjopen.bmj.com/site/about/guidelines.xhtml

weeks. With consent, participants' electronic medical records will be reviewed for data on prescriptions and consultations.

Parents will be sent regular newsletters and receive automatic emails or text reminders when their questionnaires are due. In recognition of their time and to encourage retention, parents will be offered £10 vouchers at the baseline and 16 weeks. We will also offer the child a small gift, e.g. "bee" toy, of about £5 in value.

Masking

Table 3 summarises who is masked to treatment allocation. Procedures to maintain masking to allocation will be written and followed. Researcher masking will be assessed using the Bang's blinding index.[32] Because parents, participants and treating clinicians will know the treatment allocation, un-masking procedures are not required.

Sample size

As we have four groups, we powered our sample size calculation to detect a clinically meaningful differences in six pairwise comparisons subsequent to a global test. We estimate that 416 participants (104 in each group) are required to detect a difference of 3.0 in POEM scores[12 18 19] between any two groups with 90% power and a significance level of 0.05 (after adjustment for multiple pairwise comparisons). We assumed a standard deviation (SD) of 5.5 (SD of 4.89 observed in feasibility trial[20]) to allow for greater variability in the data or smaller differences to be detected. To allow for 20% loss to follow-up, we propose recruiting 520 patients in total.

Data management

Personal data of participants' and their parents will be treated as strictly confidential and entered onto a secure administrative database stored on the University of Bristol server. Anonymised trial data will be entered onto the study's REDCap database. This system will also be used to administer online questionnaires for those who choose for online rather than paper questionnaires. The system incorporates data entry and validation rules to reduce data entry errors and management functions to facilitate auditing and data quality assurance."

Statistical methods

The analysis and presentation of the trial data will be in accordance with Consolidated Standards of Reporting Trials (CONSORT) guidelines[21 22] . A full statistical analysis plan has been developed and approved by the independent statistician on the study's trial steering committee ahead of analysis of post-randomisation data and will be made available via the study website.

Baseline characteristics of patients will be compared between the four arms by reporting summary statistics. Characteristics will be reported as means and SD, medians and inter-quartile ranges or frequencies and proportions depending on the nature of the data and its distribution. If baseline characteristics of any two treatment groups differ by more than 10% or 0.5SD then the effect of this variable on the primary outcome will be investigated in a sensitivity analysis.

Primary statistical analyses between the randomised groups will be conducted on an intention-to-treat (ITT) basis. For the primary outcome we will use linear mixed models (weekly observations, level 1, nested within participants, level 2) to explore whether there are differences in mean POEM scores between treatment groups after adjusting for baseline scores and all stratification and minimisation variables used in the randomisation. Pairwise comparisons will be conducted to

For peer review only - http://bmjopen.bmj.com/site/about/guidelines.xhtml

identify which intervention groups differed. To account for multiple testing, we will use a modified alpha of 0.0083 (0.05/6 pairwise comparisons equivalent).

Secondary outcomes will be analysed according to the data type and frequency of recording. Continuous outcomes measured at multiple time points will be analysed similarly to the primary outcome as described above. Continuous outcomes measured once after randomisation – such as EASI score – will be analysed using linear regression adjusting for baseline values where available. We will consider alternative methods should assumptions not be met.

To assess adherence to the allocated medication, for each participant, we will count the number of days of self-reported use of the allocated type of emollient and express that as a proportion of the number of days for which non-missing emollient data are available. Contamination will be assessed by calculating the proportion of days (among days where non-missing emollient data are available) where a non-allocated emollient type was used. We are unable to pre-specify what constitutes "substantial contamination", which may inform further sensitivity analyses.

Other proposed sensitivity analyses include an exploration of patterns of missing data and we will consider possible mechanisms for this. Based on these and observed data, appropriate methods for imputing missing data will be considered in sensitivity analyses. Also, should there be evidence of imbalance between treatment groups on important baseline characteristics we will conduct a regression analysis of the primary outcome adjusting additionally for these variables.

Descriptive analysis of safety endpoints will be presented both according to randomised group. Pre-specified subgroup analyses will investigate whether treatment effectiveness is modified by the following factors measured at randomisation: parent expectation; age of child at randomisation; disease severity; and eczema diagnosis. These subgroup analyses will involve incorporating interaction terms with treatment allocation to test the null hypothesis of no variation in treatment effect across subgroups. These tests are likely to be underpowered, however, therefore emphasis will be placed on the point estimates and confidence intervals generated.

Nested qualitative study

The aims of the qualitative study are firstly, to support and optimise participant recruitment and retention; and secondly, to complement, explain and aid understanding of the quantitative findings

Baseline appointment recordings: To meet the first aim, a sample of baseline appointments (at least one per recruiting researcher) will be audio-recorded and reviewed a qualitative researcher. Using a structured template, the interaction will be reviewed to ensure key information is relayed and parent understanding checked. Recommendations will be feedback individually to the relevant researcher and collectively (anonymised) to other recruiting researchers and the trial management group. Prior to the start of the baseline appointment, parents will be asked to give verbal consent for the recording, with written consent obtained at the end of the appointment.

Interviews with parents and trial participants: To meet the second aim, we will interview parents and, at their discretion, the participating children themselves, at four weeks and 16 weeks after randomisation. The design is cross-sectional, with different families interviewed at each time point. However, where particularly interesting issues emerge, we may speak to a family at both time points. Parents will indicate on the trial consent form whether they are willing to be approached for these.

For peer review only - http://bmjopen.bmj.com/site/about/guidelines.xhtml

The four-week interviews will focus on the initial use and acceptability of the assigned emollient. We will conduct up to five interviews in each trial group (total ~20), purposively sampling by: recruitment centre, age of child, eczema severity and allocated type of emollient. We will include those who have stopped using the allocated treatment or switched emollient.

The 16-week interviews will focus on the overall experience of using the assigned emollient, perceived effectiveness, planned future use of emollients and experience of taking part in the trial. The sampling criteria will be the same as for the four week interviews, with the additional criterion of intentions regarding future emollient use. We expect to achieve data saturation by conducting up to 10 interviews in each trial group (total ~40).

Interviews are expected to last between 30-60 minutes. Topic guides (including sub-topic guide for children) will be used but with flexibility to allow unanticipated issues to emerge and be further explored in later interviews. Interviews will be captured using an encrypted digital voice recorder, transcribed and anonymised to protect confidentiality.

The interview data will be analysed thematically, using a combination of deductive and inductive coding[23] and adapted techniques of constant comparison.[24] Analysis will be led by the qualitative researcher, with input from the qualitative co-applicants and trial management group. Data management and coding will be aided by use of NVivo software. Data will be compared within and across trial group, with attention to converging and diverging perspectives. The themes will be written up as interpretive summaries with illustrative verbatim quotes that represent the range of expressed views.

Monitoring, safety and audit

As the randomised treatments within this study do not differ from common usual clinical practice, risk-based monitoring will be implemented in line with a risk-assessment. Data on adverse events will be collected by parent self-report. No interim analyses are planned.

An independent Data Monitoring Committee has been established and terms of reference have been drawn up and agreed. The committee will meet at least annually, and its role is to safeguard the interests of the trial's participants, potential participants, investigators and sponsor; to assess the safety and efficacy of the trial's interventions, and to monitor the trial's overall conduct, and protect its validity and credibility.

The sponsor organisation is the University of Bristol. Adverse event reporting will be in accordance with local procedures.

The trial may be prematurely discontinued due to lack of recruitment or by the Sponsor, Chief Investigator, Regulatory Authority or Funder based on new safety information or for other reasons given by the Trial Steering Committee or Data Monitoring Committee, regulatory authority or ethics committee concerned.

PUBLIC AND PATIENT INVOLVEMENT

In 2013, the James Lind Alliance published the eczema research priorities for patients and healthcare professionals and "Which emollients are the most effective and safe in treating eczema?" emerged as one of the highest ranked uncertainties.[25]

Co-author AR is mother of children with eczema and a member of Nottingham Support Group for Carers of Children with Eczema. We have established a group of parents of children with eczema,

For peer review only - http://bmjopen.bmj.com/site/about/guidelines.xhtml

who helped develop the study and want to support our on-going work through meetings and email communication. A PPI member sits on the trial steering committee. We will use the internet and social media to promote wider patient engagement.

PPI has helped us to frame the research question around, “Which emollient to prescribe first?” for childhood eczema, acknowledging that individuals differ in their experiences of effectiveness and tolerability of different emollients. It has also gave us a clear steer that including a non-emollient group would be unacceptable to many families; favoured POEM as the primary outcome; and highlighted how emollient use may be a “trade off” between effectiveness and acceptability.

On-going PPI involvement has informed both qualitative and quantitative data collection and helps ensure that the study continues to focus on delivering clinically important outcomes that are meaningful to patients.[26]

ETHICS AND DISSEMINATION

Research ethics approval

The study has been reviewed by the Health Research Authority and given a favourable opinion by the NHS REC (South West - Central Bristol Research Ethics Committee 17/SW/0089).

Protocol amendments

Any amendments to the Protocol will be reported accordingly to the regulatory bodies, with a copy of the current protocol (version 6.0 currently) available for download from the study website. Amendments to date are listed in appendix 1.

Consent and assent

Written consent for taking part in the trial will be received by a researcher from the parent or guardian of the participant at their baseline appointment. For children approximately 7 years and older, the option of providing assent will be offered alongside parental consent.

Confidentiality and access to data

The database and randomisation system will protect patient information in line with the data protection legislation. Trial staff will ensure that participants’ anonymity is maintained through protective and secure handling and storage of patient information at the lead centre. The Chief Investigator will have access to and act as custodian of the full dataset.

Ancillary and post-trial care

After the 16-week primary outcome period, participants will be free to change their emollient if they wish. Conversely, they will be able to continue with their allocated emollient after they have completed follow-up.

Dissemination and data sharing

A series of stakeholder meetings will raise study awareness amongst and share progress and findings with policy makers, voluntary groups, clinicians, patients, families. Study progress, outputs and a summary of findings will be made available via a study website and Twitter account; and summaries distributed to participating families and GP surgeries. Findings will be submitted for presentation at conferences and written up for publication in a peer-reviewed journal(s), which may include integration of the quantitative and qualitative findings. The International Committee of Medical

For peer review only - http://bmjopen.bmj.com/site/about/guidelines.xhtml

For peer review only

Journal Editors has criteria for authorship will be observed and no professional writers will be employed.

No later than three years after the completion of the study, we will deposit a deidentified data set in an appropriate data archive.

DISCUSSION

Factors that may influence patient preference for different types of emollient include disease severity, body site, cosmetic acceptability of the product, season/climate and packaging.[27] Cultural factors may also influence choice and use.[28] NICE recommends patients try different emollients in the clinic before choosing.[5] This approach is not practical in primary care, and even in specialist settings the range of emollients available to try can be arbitrary – restricted by local formularies and influenced of pharmaceutical companies. Therefore, most patients consulting in primary care are unaware of differences between emollients; and many primary care clinicians will be unable to advise on grounds other than consistency or simple unit cost.

Some emollients are decades old and it has not been in the interest of manufacturers to submit their products in a head-to-head comparison with others in a clinical trial. In BEE, we are independently evaluating in a pragmatic trial, using a validated patient-reported primary outcome, the effectiveness of the four types of emollients commonly prescribed for children with eczema. In accordance with the recommendations of HOME (Harmonising Outcome Measures in Eczema), POEM and EASI will be used to measure patient-reported symptoms and clinical signs, respectively.[29]

Participants are unmasked, so by knowing which emollient they're using may bias assessment of emollient effectiveness. However, we have chosen a patient-reported outcome as the primary outcome because symptoms of eczema are more important to families of than objective measures which are based on skin appearance.[11] We will minimise the potential for performance bias by ensuring that at the point of consent parents are willing to use any of the four emollients for the first 16 weeks. We will also measure at baseline parent opinion regarding the four different study emollients, and in a sub-group analysis explore whether reported effectiveness is linked to high/low prior expectations of effectiveness. The collection of an objective measure of eczema severity (EASI) by a masked researcher as a secondary outcome allows us to examine outcomes in relation to signs of eczema. Subject to additional funding, we plan to undertake a full economic evaluation to determine the cost-effectiveness of the four emollient types.

Recruitment started in January 2018 and follow-up of the last participant is scheduled by February 2020. Findings from the BEE study, comparing the clinical effectiveness and acceptability of commonly used different emollients, will provide evidence upon which clinicians and carers/patients can decide which emollient to try first. Our aim is not to reduce choice, but to reduce uncertainty and the consequences of "trial and error" prescribing. Smarter prescribing will help prescribers and carers gain "control" over the eczema more quickly, reduce frustration and inconvenience for families, and potentially produce cost savings to the NHS through cost-effective prescribing and fewer repeat consultations to change emollients.

For peer review only - http://bmjopen.bmj.com/site/about/guidelines.xhtml

STATEMENTS

Acknowledgements

The BEE study:

- is hosted by Bristol, North Somerset and South Gloucestershire (BNSSG) CCG Research and Evidence.
- was developed with support from UK Dermatology Clinical Trials Network (UK DCTN). The UK DCTN is grateful to the British Association of Dermatologists and the University of Nottingham for financial support of the Network.
- was designed and is being delivered in collaboration with the Bristol Randomised Trials Collaboration (BRTC), a UKCRC registered clinical trials unit which, as part of the Bristol Trials Centre, is in receipt of National Institute for Health Research CTU support funding.

Study data are collected and managed using REDCap[30] hosted at the University of Bristol.

We are grateful to the members of the following trial oversight committees:

- Trial steering committee: Professor Richard McManus (chair), Dr Ben Carter (medical statistician), Professor Joanne Protheroe, Dr Sariqa Wagley and Dr Andrew Moore.
- Data monitoring committee members: Dr John Ingram (chair), Dr Catriona Keerie (medical statistician), Dr Chin Whybrew.

Declaration of interests

No interests to declare.

Funding

The study is funded by National Institute for Health Research (NIHR) HTA programme (Ref: 15/130/07). MJR is funded by an NIHR Post-Doctoral Research Fellowship (PDF-2014-07-013). JPB was supported by the National Institute for Health Research (NIHR) Collaboration for Leadership in Applied Health Research and Care West (CLAHRC West) at University Hospital Bristol NHS Foundation Trust. The views expressed in this publication are those of the authors and not necessarily those of the NHS, the National Institute for Health Research or the Department of Health and Social Care.

Author contributions

MR conceived the study idea and developed the initial study design with KT, HCW, MS, SJM, JPB, AR, ARGS and ADH. TJB, JT and AL helped further develop the initial proposal and LE/SW assisted with the study protocol. Specific advice on trial design and medical statistics was given by SJM, ESa and JT; on the nested qualitative study by ARGS, JPB and ESu; and on the collection of health economic data by KG. Led by MJR, all the authors contributed to the drafting of the study protocol and approved the final manuscript.

The TMG would like to thank Dr Sandra Hollinghurst for her advice on the collection of data relevant to health economics; and Mr Paul Roy and Ms Rachel Avery of BNSSG CCG Research and Evidence for their support in the delivery of the trial and dissemination of findings.

For peer review only - http://bmjopen.bmj.com/site/about/guidelines.xhtml

Study website and social media

www.bristol.ac.uk/bee-study and Twitter: @bee-study

REFERENCES

1. Williams HC. Atopic dermatitis: The epidemiology, causes and prevention of atopic eczema. Cambridge: Cambridge University Press 2000.
2. Ben-Gashir M, Seed P, Hay R. Quality of life and disease severity are correlated in children with atopic dermatitis. *British Journal of Dermatology* 2004;150(2):284-90.
3. Johansson S, Bieber T, Dahl R, et al. Revised nomenclature for allergy for global use: Report of the Nomenclature Review Committee of the World Allergy Organization, October 2003. *J Allergy Clin Immunol* 2004;113:832-36.
4. Emerson RM, Williams HC, Allen BR. Severity distribution of atopic dermatitis in the community and its relationship to secondary referral. *British Journal of Dermatology* 1998;139:73-76.
5. NICE. CG057: Atopic eczema in children - management of atopic eczema in children from birth up to the age of 12 years. London: RCOG Press, 2007.
6. Van Zuuren EJ, Fedorowicz Z, Christensen R, et al. Emollients and moisturisers for eczema. *Cochrane Database of Systematic Reviews* 2017(2) doi: 10.1002/14651858.CD012119.pub2.
7. Chan JP, Boyd G, Quinn PA, et al. Emollient prescribing formularies in England and Wales: a cross-sectional study. *BMJ Open* 2018;8(6) doi: 10.1136/bmjopen-2018-022009
8. Santer M, Burgess H, Yardley L, et al. Experiences of carers managing childhood eczema and their views on its treatment: a qualitative study. *British Journal of General Practice* 2012;62(597):e261-e67. doi: 10.3399/bjgp12X636083
9. Le Roux E, Powell K, Banks JP, et al. GPs' experiences of diagnosing and managing childhood eczema: a qualitative study in primary care. *British Journal of General Practice* 2018;68(667):e73-e80. doi: 10.3399/bjgp18X694529
10. Charman C, Venn A, Ravenscroft J, et al. Translating Patient-Oriented Eczema Measure (POEM) scores into clinical practice by suggesting severity strata derived using anchor-based methods. *Br J Dermatol* 2013;169(6):1326-32. doi: 10.1111/bjd.12590
11. Charman CR, Venn AJ, Williams HC. The Patient-Oriented Eczema Measure: Development and Initial Validation of a New Tool for Measuring Atopic Eczema Severity From the Patients' Perspective. *Archives of Dermatology* 2004;140(12):1513-19.
12. Gaunt DM, Metcalfe C, Ridd M. The Patient-Oriented Eczema Measure in young children: responsiveness and minimal clinically important difference. *Allergy* 2016;71:1620-25. doi: 10.1111/all.12942
13. Schmitt J, Langan S, Williams HC. What are the best outcome measurements for atopic eczema? A systematic review. *Journal of Allergy and Clinical Immunology* 2007;120(6):1389-98. doi: 10.1016/j.jaci.2007.08.011
14. Stevens KJ, Brazier JE, McKenna SP, et al. The development of a preference-based measure of health in children with atopic dermatitis. *British Journal of Dermatology* 2005;153(2):372-77.
15. Lawson V, Lewis-Jones MS, Finlay AY, et al. The family impact of childhood atopic dermatitis: the Dermatitis Family Impact Questionnaire. *British Journal of Dermatology* 1998;138(1):107-13. doi: 10.1046/j.1365-2133.1998.02034.x
16. Stevens K. Assessing the performance of a new generic measure of health-related quality of life for children and refining it for use in health state valuation. *Applied Health Economics and Health Policy* 2011;9(3):157-69.
17. Stevens K, Katherine S. Valuation of the Child Health Utility 9D Index. *Pharmacoeconomics* 2012;30:729-47.

For peer review only - http://bmjopen.bmj.com/site/about/guidelines.xhtml

18. Schram ME, Spuls P, Leeflang MMG, et al. EASI, (objective) SCORAD and POEM for atopic eczema: responsiveness and minimal clinically important difference. *Allergy* 2012;67(1):99-106.
19. Howells L, Ratib S, Chalmers J R, Bradshaw L, Thomas K S. How should minimally important change scores for the Patient-Oriented Eczema Measure be interpreted? A validation using varied methods. *British Journal of Dermatology* 2018;178(5):1135-42. doi: 10.1111/bjd.16367
20. Ridd MJ, Garfield K, Gaunt DM, et al. Choice of Moisturiser for Eczema Treatment (COMET): feasibility study of a randomised controlled parallel group trial in children recruited from primary care. *BMJ Open* 2016;6(11) doi: 10.1136/bmjopen-2016-012021
21. Calvert M, Blazeby J, Altman DG, et al. Reporting of patient-reported outcomes in randomized trials: The consort pro extension. *JAMA* 2013;309(8):814-22. doi: 10.1001/jama.2013.879
22. Zwarenstein M, Treweek S, Gagnier JJ, et al. Improving the reporting of pragmatic trials: an extension of the CONSORT statement. *BMJ* 2008;337 doi: 10.1136/bmj.a2390
23. Braun V, Clarke V. Using thematic analysis in psychology. *Qual Res Psychol* 2011;3 doi: 10.1191/1478088706qp063oa
24. Strauss AL, Corbin J. Basic of qualitative research: grounded theory procedures and techniques. London: Sage 1998.
25. Batchelor JM, Ridd MJ, Clarke T, et al. The Eczema Priority Setting Partnership: a collaboration between patients, carers, clinicians and researchers to identify and prioritize important research questions for the treatment of eczema. *British Journal of Dermatology* 2013;168(3):577-82. doi: 10.1111/bjd.12040
26. Sutton E, Dawson S, Roberts A, et al. Patient and public involvement (PPI) in the Best Emollient for Eczema (BEE) Study. South West Meeting of the Society for Academic Primary Care. Southampton, 2019.
27. Moncrieff G. Use of emollients in dry skin conditions: consensus statement. *Clin Exp Dermatol* 2013;38:231-38.
28. Santer M, Muller I, Yardley L, et al. Parents' and carers' views about emollients for childhood eczema: qualitative interview study. *BMJ Open* 2016;6(8) doi: 10.1136/bmjopen-2016-011887
29. Schmitt J, Spuls P, Boers M, et al. Towards global consensus on outcome measures for atopic eczema research: results of the HOME II meeting. *Allergy* 2012;67:1111-17. doi: 10.1111/j.1398-9995.2012.02874.x
30. Harris PA, Taylor R, Thielke R, et al. Research electronic data capture (REDCap)—A metadata-driven methodology and workflow process for providing translational research informatics support. *Journal of Biomedical Informatics* 2009;42(2):377-81. doi: https://doi.org/10.1016/j.jbi.2008.08.010

For peer review only - http://bmjopen.bmj.com/site/about/guidelines.xhtml

FIGURE

Figure 1: Overview of participant pathway through the study

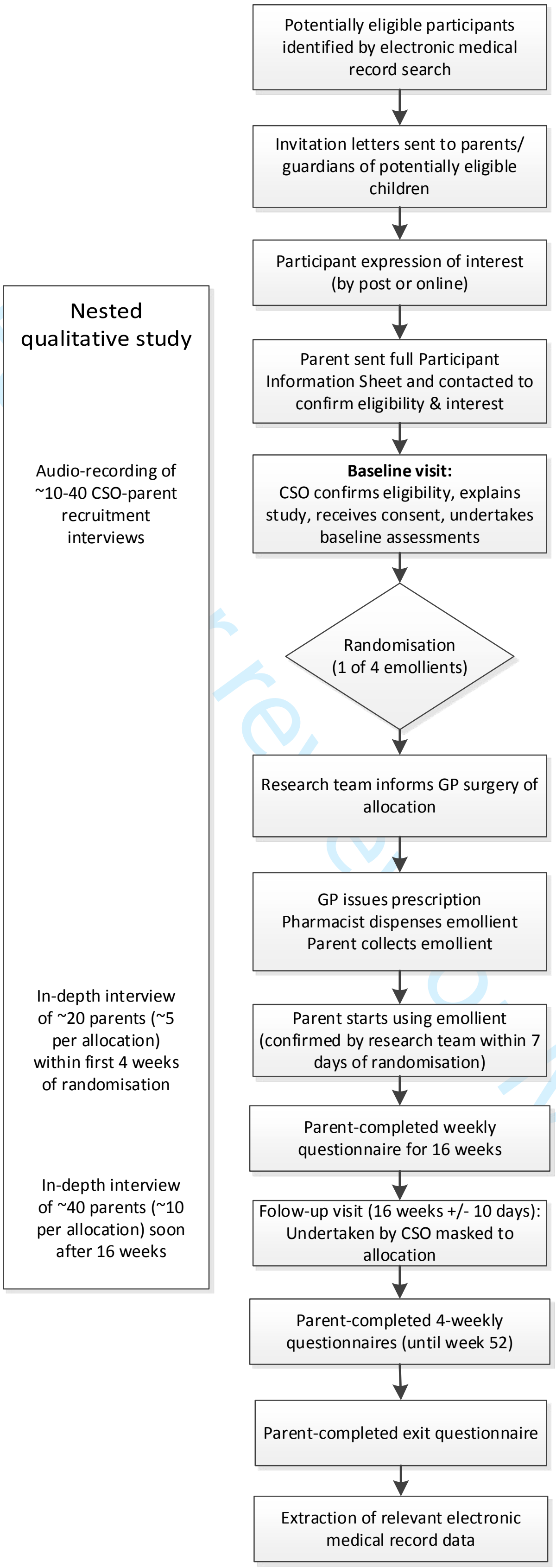

For peer review only - http://bmjopen.bmj.com/site/about/guidelines.xhtml

BOX

Box: Participant eligibility criteria

Inclusion criteria

Children must:

- be aged between 6 months and less than 12 years of age
- have eczema diagnosed by an appropriately qualified healthcare professional (registered doctor, nurse or health visitor)
- mild eczema or worse (POEM score>2 within previous 28 days)

The person giving consent must:

- have parental responsibility for the participant
- be willing to use the randomly allocated emollient type as the only leave-on emollient for 16 weeks.

Exclusion criteria

Child:

- known sensitivity to study emollients or their constituents
- participating in another research study currently or in the last four months
- any other known adverse medical or social circumstance that would make invitation to the study inappropriate (as determined by GP practice staff)

The person giving consent:

- unable to give informed consent
- insufficient written English to complete outcome measures

For peer review only

For peer review only - http://bmjopen.bmj.com/site/about/guidelines.xhtml

TABLES

Table 1: Rules for exclusion/inclusion of different types of emollients

**Type of emollient**		**Lotion**	**Cream**	**Gel**	**Ointment**
Rules/group shared characteristics	Exclusion	Antimicrobials or urea			
	Inclusion	Paraffin-based			
		Glycerol containing only	No humectant or lanolin	Does not contain povidine	No additives
Example formulary emollients from each group†		Cetraben lotion, QV lotion and Diprobase lotion	Diprobase cream, Epimax cream, Aquamax cream, Zerobase cream and AproDerm cream	Doublebase gel, Isomol gel, Zerodouble gel, AproDerm gel and MyriBase gel	Diprobase ointment, Emulsifying ointment BP, White soft/Liquid paraffin 50/50 ointment, Paraffin White soft ointment and Paraffin Yellow soft ointment

† Membership will be monitored and may change over time, keeping within the inclusion and exclusion criteria for each group.

For peer review only

For peer review only - http://bmjopen.bmj.com/site/about/guidelines.xhtml

Table 2: Schedule of enrolment, interventions and assessments

	Study period																												Close-out
	Enrolment	Allocation	Post-allocation																										
	S	V_0	Participant questionnaires															V_1	Participant questionnaires									
Week		0	1	2	3	4	5	6	7	8	9	10	11	12	13	14	15	16	16	20	24	28	32	36	40	44	48	52	
Parent completed																													
Screening questionnaire	●																												
Opinion about emollients		●																											
POEM	●	●	●	●	●	●	●	●	●	●	●	●	●	●	●	●	●	●		●	●	●	●	●	●	●	●	●	
Eczema pain & bother		●				●				●				●				●											
Use of treatments for eczema		●	●	●	●	●	●	●	●	●	●	●	●	●	●	●	●	●		●	●	●	●	●	●	●	●	●	
Adverse events			●	●	●	●	●	●	●	●	●	●	●	●	●	●	●	●		●	●	●	●	●	●	●	●	●	
Consultations (non-EMR)						●				●				●				●		●	●	●	●	●	●	●	●	●	
Personal costs						●				●				●				●		●	●	●	●	●	●	●	●	●	
DFI		●																●										●	
ADQoL		●						●										●										●	
CHU-9D		●						●										●										●	
Satisfaction with emollient																		●											
Study experiences																												●	
Researcher administered																													
Demographics and history		●																											
UK diagnostic criteria for AD		●																											
EASI		●																	●										
EMR notes review																													●
**Nested qualitative study**																													
Audio-recording		○																											
Round one interviews				←	○	→																							
Round two interviews																			← ○	→									

● = all participants; ○ = sample of participants

S: screening stage (responses to written invitation letters and people responding to opportunistic invites); V_0 and V_1: research face-to-face baseline & follow-up visits

POEM: Patient Orientated Eczema Measure; † bother score, itch intensity, parent global assessment; ADQoL: Atopic Dermatitis Quality of Life; IDQoL: Infant Dermatitis Quality of Life; CHU-9D: Children's Health Utility 9D; GAD-7: Generalised Anxiety Disorder 7; EASI: Eczema Area Severity Index; AD: Atopic Dermatitis. EMR: Electronic Medical Record

For peer review only - http://bmjopen.bmj.com/site/about/guidelines.xhtml

Table 3: Masking to treatment allocation

Individual(s)	Status
Participating children, their parents and any treating clinician	Unmasked: the allocated emollient is prescribed by the participant's GP and issued by local pharmacy as in usual care
Clinical Trials Unit (CTU) database staff, Trial Coordinators and Trial Administrator and Qualitative RA	Unmasked: CTU staff will maintain the randomisation database. The trial coordinator/administrator will randomise participants and be the initial point of contact for all enquiries relating to issues with the emollients.
Qualitative team (Drs Sutton, Heawood and Banks)	Unmasked: Participants will be sampled based on emollient allocation/use and during the interviews the qualitative researcher will specifically ask about the different emollient types.
Junior statistician (Ms Sanderson)	Unmasked: The junior statistician was initially masked knowing only an anonymised code for the different treatment groups. After approval of the statistical analysis plan, she was unmasked to permit preparation and discussion of unmasked data with the data monitoring committee.
Trial Manager and Chief Investigator	Masked: The Trial Manager was masked prior to the writing of the statistical analysis plan but is unmasked on an individual participant basis, when required to undertake randomisations and deal with potential serious adverse events. The Chief Investigator will only be unmasked in the event of a serious adverse event.
Other Trial Management Group members: Dr MacNeill (senior statistician), Dr Santer & Prof Thomas (PIs), Ms Barrett (pharmacist), Dr Lane & Dr Taylor (CTU), Professors Hay & Williams (senior researchers), Ms Kirsty Garfield (Health economist), Dr Baxter (knowledge mobilisation), Mrs Roberts (PPI)	Masked: procedures will be put in place to maintain masking both within and outside of project meetings.
Researchers	Masked: masking of researchers undertaking baseline and 16-week visits will be monitored by means of self-report.

For peer review only - http://bmjopen.bmj.com/site/about/guidelines.xhtml

Appendix 1: Administrative information

Title

The Best Emollient for Eczema (BEE) trial: a randomised trial comparing the effectiveness of four types of commonly prescribed emollients for children with eczema

Trial registration number

ISRCTN: ISRCTN84540529 (Date registered: 05/06/2017)

World Health Organization Trial Registration Data Set

Data category	Information
Primary registry and trial identifying number	ISRCTN84540529
Date of registration in primary registry	05.06.2017
Secondary identifying numbers	EudraCT: 2017-000688-34
Source(s) of monetary or material support	NIHR Health Technology Assessment (HTA) 15/130/07
Primary sponsor	University of Bristol
Secondary sponsor(s)	Not applicable
Contact for public queries	bee-study@bristol.ac.uk, 0117 928 7351
Contact for scientific queries	Dr Matthew Ridd FRCGP PhD, m.ridd@bristol.ac.uk, 0117 331 4557
Public title	Best Emollients for Eczema (BEE) study
Scientific title	The Best Emollient for Eczema (BEE) trial: a randomised trial comparing the effectiveness of four types of commonly prescribed emollients for children with eczema
Countries of recruitment	England
Health condition(s) or problem(s) studied	Childhood eczema
Intervention(s)	Lotion, cream, gel or ointment as the only leave-on emollient for 16 weeks with directions to apply twice daily and as required.
Key inclusion and exclusion criteria	CHILD<br>Inclusion criteria:<br>1. Aged between 6 months and less than 12 years of age<br>2. Have eczema diagnosed by an appropriately qualified healthcare professional (registered doctor, nurse or health visitor)<br>3. Have mild eczema or worse (POEM score>2)<br><br>Exclusion criteria:<br>1. Known sensitivity to study emollients or their constituents<br>2. Participating in another research study currently or in the last four

For peer review only

	months<br>3. Any other known adverse medical or social circumstance that would make invitation to the study inappropriate (as determined by GP practice staff)<br><br>PERSON GIVING CONSENT:<br>Inclusion criteria:<br>1. Have parental responsibility for the participant<br>2. Willing to use the randomly allocated emollient as the only leave-on emollient for 16 weeks.<br><br>Exclusion criteria:<br>1. Unable to give informed consent<br>2. Insufficient written English to complete outcome measures.
Study type	Intervention
Date of first enrolment	19 January 2018
Target sample size	520
Recruitment status	Recruiting
Primary outcome(s)	Eczema symptoms, measured using POEM over 16 weeks
Key secondary outcomes	Eczema symptoms, measured using POEM over 52 weeks; eczema signs, measured using EASI; eczema 'bother' score; itch intensity score; parent global assessment of eczema; other possible symptoms of food allergy; UK diagnostic criteria for atopic dermatitis; main carer anxiety, measured using GAD-7; diet of child and/or mother if child being breastfed by her; adverse events; child and family quality of life, measured using ADQoL, CHU-9D and IDQoL; satisfaction with trial processes, procedures and paperwork; health services utilisation; out-of-pocket expenses/time off work.

Protocol version and history of amendments

The current version of the protocol is 6.0 (10.06.19). Previous protocols and amendments are as below:

Version		Notes
**Number**	**Date**	
1.0	21.03.2017	Submitted for approval (March 2017) and approval received from REC, MHRA and HRA.
2.0	27.06.2017	Title page: ISRCTN, NHS REC, and NIHR portfolio numbers added; 10.3: Clarification of eligibility confirmation; 10.6: "Blinding to treatment allocation" table amended to reflect changes in research team/processes to minimize un-blinding of TMG members, in accordance with TS/DM-C recommendation; 12.3: Clarification that first set of interviews will be with participants during their first four weeks in the study, not during the first four weeks of the life of the trial itself; 19.2: clarity to TS/DM-C composition/roles; 14.3: clarification about who makes decisions regarding causality of adverse events/reactions.
3.0	03.08.2017	Clarification that screening POEM must be within 28 days of recruitment. Removal of signature page to separate document.

For peer review only - http://bmjopen.bmj.com/site/about/guidelines.xhtml

4.0	03.11.17	Amendment to the intervention, from 4 specific emollients, to type of emollient. Correction of minor typos. Clarification of Safety reporting section. Update to "Timetable and milestones" to reflect delayed start to internal pilot.
5.0	01.08.18	Change "Bristol CCG" to "Bristol, North Somerset and South Gloucestershire CCG" to reflect merger/name change (page 6); changes to blinding arrangements (**Error! Reference source not found.**) and removal of reference to "Ms Jameson", former CAPC PPI&E coordinator who was never a TMG member and has subsequently left (page 27/28); update to section 19.2 (Oversight committees) to describe separate TSC and DMC created at request of funder after approval of protocol 4.0; other minor grammatical/style changes/corrections.
6.0	10.06.19	Updated references to timelines throughout to reflect 38-months recruitment and follow-up / 50-month total study duration. Insertion of paragraph on participant communication (section 10.8, Participant stipends and communication). Replace Avon Primary Care Research Collaboration logo with BNSSG CCG logo. Replace any reference to blind, blinded or blinding with masked or masking. Extra information for parents of study participants in order to bring study in-line with the EU General Data Protection Regulations 2018. Minor changes to titles/postal addresses.

Funding

NIHR Health Technology Assessment (HTA) 15/130/07

Contributorship

See main manuscript

Sponsor contact information

Trial sponsor: University of Bristol
Sponsor's reference: 2738
Contact name: Dr Rachel Davies
Address: Research Enterprise Development, One Cathedral Square, Bristol BS1 5DD
Email: research-governance@bristol.ac.uk
Telephone: 0117 428 4011

Role of study sponsor and funder

The funder and sponsor had no role in the design of this study and will not have any role during its execution, analyses, interpretation of the data, or decision to submit results.

Committees

The study is hosted by BNSSG CCG, and will be delivered by the University of Bristol, in collaboration with partners at University of Nottingham, University of Southampton. The Universities of Nottingham and Southampton will be recruiting centres, with Professor Kim Thomas and Dr Miriam Santer as the Principal Investigators, respectively.

For peer review only - http://bmjopen.bmj.com/site/about/guidelines.xhtml

The Trial Management Group comprises all investigators, the trial manager, research and administrative staff, the trials unit and patient/public representative. Members will contribute to the trial in the following ways: trial design and methods; participant recruitment and trial conduct; trial management; trial logistics and cost management; economic evaluation; qualitative study statistical data analysis; and publication. The Trial Management Group will meet on a regular basis to oversee the management of the trial. Meetings will be face-to-face with teleconference facilities for TMG members who are unable to be present.

This study was designed and is being delivered in collaboration with the Bristol Randomised Trials Collaboration, a UKCRC registered clinical trials unit which, as part of the Bristol Trials Centre, is in receipt of National Institute for Health Research Clinical Trials Unit support funding.

Because this is a low-risk trial, the funder originally agreed that the roles of both guiding the TMG and monitoring trial data will be undertaken by a single joint committee, the Trial Steering/Data Monitoring Committee. However, because of changes implemented in version 4.0 of the protocol, the funder requested that separate Trial Steering and Data Monitoring Committees be established.

The Trial Steering Committee will provide overall supervision of the trial on behalf of the funder. Terms of reference have been drawn up and agreed with members, which comprises four independent members: a chairperson, an academic, a biostatistician and a patient representative (parent of child with eczema). There is one additional non-independent member who is a qualitative researcher. Non-independent members will not have any voting rights. The Trial Steering Committee will meet at least four times over the course of the study, including one which will coincide with the end of the internal pilot and a final meeting, when analysis is almost complete and the final report is being prepared.

The Data Monitoring Committee will safeguard the interests of the trial's participants, potential participants, investigators and sponsor; to assess the safety and efficacy of the trial's interventions, and to monitor the trial's overall conduct, and protect its validity and credibility. Terms of reference have been drawn up and agreed with members, which comprises three independent members: a chairperson, a biostatistician and GP with specialist interest in dermatology. The Data Monitoring Committee will meet at least annually: only committee members and the junior statistician should be present in closed sessions; open sessions will be attended by those at the closed session, plus the CI and possibly representatives of the sponsor or funder, and a trial unit representative.

For peer review only

For peer review only - http://bmjopen.bmj.com/site/about/guidelines.xhtml

Best Emollient for Eczema (BEE) Study

Parent/Carer Consent Form

Initial box

1. I confirm that I have read and understand the Participant Information Sheet dated 03.11.2017 (version 3.0) for the above study. I have had the opportunity to consider the information, ask questions and have had these questions answered satisfactorily. ☐
2. I understand that participation is voluntary and that we are free to withdraw at any time without giving any reason, without my child's medical care or legal rights being affected. ☐
3. I understand that after the study ends, the data collected will be made "open data". I understand that this means the anonymised data will be publicly available and may be used for purposes not related to this study, and it will not be possible to identify me from these data. ☐
4. I understand that relevant sections of my child's medical notes and all information collected for this research may be reviewed by the study team, from regulatory authorities or from the NHS Trust for the purpose of ensuring that the research is conducted appropriately. I give permission for these individuals to access my child's records as appropriate. ☐
5. I give permission for researchers working on this study to have access to my child's medical records for the purposes of collecting information relevant to the aims of this study. ☐
6. I give consent for the data collected in this trial to be used in future ethically approved studies on the understanding that all information will continue to be kept securely and remain confidential. ☐
7. I give consent to be contacted by a member of the research team with a view to being interviewed about my experiences of emollients and taking part in BEE. I understand that I will be given more information first, I can decide later about taking part in this, I will be asked to give further consent for taking part in interviews and that I may not be contacted at all. ☐
8. *For those asked to take part in audio-recording of recruitment visit only:* I agree to have my recruitment visit audio-recorded, including anything my child may say. I agree to data from my audio-recorded interview being transferred to and retained by the Universities of Bristol, Southampton and Nottingham for training, teaching and research purposes, now and in the future. ☐
9. I agree for my child to take part in the above-named study. ☐

________________________ ______________

Name of Participant (Child) Participant ID

Funded by NHS National Institute for Health Research

The research was funded by the NIHR Health Technology Assessment (HTA) Programme. The views expressed are those of the author(s) and not necessarily those of the NHS, the NIHR or the Department of Health.

For peer review only - http://bmjopen.bmj.com/site/about/guidelines.xhtml

University of BRISTOL · UNIVERSITY OF Southampton · The University of Nottingham UNITED KINGDOM · CHINA · MALAYSIA · Bristol Randomised Trials Collaboration

Name of Parent/Guardian	Signature	Date
Name of person receiving consent	Signature	Date

For peer review only

Funded by NHS National Institute for Health Research

The research was funded by the NIHR Health Technology Assessment (HTA) Programme. The views expressed are those of the author(s) and not necessarily those of the NHS, the NIHR or the Department of Health.

For peer review only - http://bmjopen.bmj.com/site/about/guidelines.xhtml

Best Emollient for Eczema (BEE) Study

Assent Form for Children

(Assent means you are agreeing to join this study)

Please circle Yes or No for each question

1.	I have read the leaflet that explains about the BEE study.	Yes	No
2.	I have been able to ask questions about it.	Yes	No
3.	I understand what the study is all about.	Yes	No
4.	I understand that I do not have to take part if I do not want to.	Yes	No
5.	I can change my mind and I do not have to say why.	Yes	No
6.	I agree to take part in the study.	Yes	No

Name of Participant (Child) ____________ Signature ____________ Participant ID ____________

Name of person receiving assent ____________ Signature ____________ Date ____________

Funded by NHS National Institute for Health Research

The research was funded by the NIHR Health Technology Assessment (HTA) Programme. The views expressed are those of the author(s) and not necessarily those of the NHS, the NIHR or the Department of Health.

For peer review only - http://bmjopen.bmj.com/site/about/guidelines.xhtml

STANDARD PROTOCOL ITEMS: RECOMMENDATIONS FOR INTERVENTIONAL TRIALS

SPIRIT 2013 Checklist: Recommended items to address in a clinical trial protocol and related documents*

Section/item	ItemNo	Description	Location
**Administrative information**			
Title	1	Descriptive title identifying the study design, population, interventions, and, if applicable, trial acronym	Page 1
Trial registration	2a	Trial identifier and registry name. If not yet registered, name of intended registry	Page 2
	2b	All items from the World Health Organization Trial Registration Data Set	Appendix 1
Protocol version	3	Date and version identifier	Page 9 & Appendix 1
Funding	4	Sources and types of financial, material, and other support	Page 11
Roles and responsibilities	5a	Names, affiliations, and roles of protocol contributors	Page 1
	5b	Name and contact information for the trial sponsor	Appendix 1
	5c	Role of study sponsor and funders, if any, in study design; collection, management, analysis, and interpretation of data; writing of the report; and the decision to submit the report for publication, including whether they will have ultimate authority over any of these activities	Appendix 1
	5d	Composition, roles, and responsibilities of the coordinating centre, steering committee, endpoint adjudication committee, data management team, and other individuals or groups overseeing the trial, if applicable (see Item 21a for data monitoring committee)	Page 11 & Appendix 1
**Introduction**			

For peer review only

Background and rationale	6a	Description of research question and justification for undertaking the trial, including summary of relevant studies (published and unpublished) examining benefits and harms for each intervention	Page 3
	6b	Explanation for choice of comparators	Pages 3 & 4
Objectives	7	Specific objectives or hypotheses	Pages 3 & 4
Trial design	8	Description of trial design including type of trial (eg, parallel group, crossover, factorial, single group), allocation ratio, and framework (eg, superiority, equivalence, noninferiority, exploratory)	Page 4
**Methods: Participants, interventions, and outcomes**			
Study setting	9	Description of study settings (eg, community clinic, academic hospital) and list of countries where data will be collected. Reference to where list of study sites can be obtained	Page 4
Eligibility criteria	10	Inclusion and exclusion criteria for participants. If applicable, eligibility criteria for study centres and individuals who will perform the interventions (eg, surgeons, psychotherapists)	Page 4/Box
Interventions	11a	Interventions for each group with sufficient detail to allow replication, including how and when they will be administered	Pages 4 & 5, Table 1
	11b	Criteria for discontinuing or modifying allocated interventions for a given trial participant (eg, drug dose change in response to harms, participant request, or improving/worsening disease)	Page 5
	11c	Strategies to improve adherence to intervention protocols, and any procedures for monitoring adherence (eg, drug tablet return, laboratory tests)	Pages 5 & 7
	11d	Relevant concomitant care and interventions that are permitted or prohibited during the trial	Page 5

For peer review only - http://bmjopen.bmj.com/site/about/guidelines.xhtml

Outcomes	12	Primary, secondary, and other outcomes, including the specific measurement variable (eg, systolic blood pressure), analysis metric (eg, change from baseline, final value, time to event), method of aggregation (eg, median, proportion), and time point for each outcome. Explanation of the clinical relevance of chosen efficacy and harm outcomes is strongly recommended	Page 5 and Table 2
Participant timeline	13	Time schedule of enrolment, interventions (including any run-ins and washouts), assessments, and visits for participants. A schematic diagram is highly recommended (see Figure)	Page 5, Figure 1 & Table 2
Sample size	14	Estimated number of participants needed to achieve study objectives and how it was determined, including clinical and statistical assumptions supporting any sample size calculations	Page 6
Recruitment	15	Strategies for achieving adequate participant enrolment to reach target sample size	Pages 4 & 7
**Methods: Assignment of interventions (for controlled trials)**			
Allocation:			
Sequence generation	16a	Method of generating the allocation sequence (eg, computer-generated random numbers), and list of any factors for stratification. To reduce predictability of a random sequence, details of any planned restriction (eg, blocking) should be provided in a separate document that is unavailable to those who enrol participants or assign interventions	Page 4
Allocation concealment mechanism	16b	Mechanism of implementing the allocation sequence (eg, central telephone; sequentially numbered, opaque, sealed envelopes), describing any steps to conceal the sequence until interventions are assigned	Page 4 & Table 3
Implementation	16c	Who will generate the allocation sequence, who will enrol participants, and who will assign participants to interventions	Page 4, Figure 1, Table 3
Blinding (masking)	17a	Who will be blinded after assignment to interventions (eg, trial participants, care providers, outcome assessors, data analysts), and how	Page 6 & Table 3

	17b	If blinded, circumstances under which unblinding is permissible, and procedure for revealing a participant's allocated intervention during the trial	N/A
**Methods: Data collection, management, and analysis**			
Data collection methods	18a	Plans for assessment and collection of outcome, baseline, and other trial data, including any related processes to promote data quality (eg, duplicate measurements, training of assessors) and a description of study instruments (eg, questionnaires, laboratory tests) along with their reliability and validity, if known. Reference to where data collection forms can be found, if not in the protocol	Page 5, Table 2
	18b	Plans to promote participant retention and complete follow-up, including list of any outcome data to be collected for participants who discontinue or deviate from intervention protocols	Page 5
Data management	19	Plans for data entry, coding, security, and storage, including any related processes to promote data quality (eg, double data entry; range checks for data values). Reference to where details of data management procedures can be found, if not in the protocol	Page 6
Statistical methods	20a	Statistical methods for analysing primary and secondary outcomes. Reference to where other details of the statistical analysis plan can be found, if not in the protocol	Pages 6 & 7
	20b	Methods for any additional analyses (eg, subgroup and adjusted analyses)	Pages 6 & 7
	20c	Definition of analysis population relating to protocol non-adherence (eg, as randomised analysis), and any statistical methods to handle missing data (eg, multiple imputation)	Page 7
**Methods: Monitoring**			
Data monitoring	21a	Composition of data monitoring committee (DMC); summary of its role and reporting structure; statement of whether it is independent from the sponsor and competing interests; and reference to where further details about its charter can be found, if not in the protocol. Alternatively, an explanation of why a DMC is not needed	Pages 8 & 11, Appendix 1

	21b	Description of any interim analyses and stopping guidelines, including who will have access to these interim results and make the final decision to terminate the trial	Page 8 and Appendix 1
Harms	22	Plans for collecting, assessing, reporting, and managing solicited and spontaneously reported adverse events and other unintended effects of trial interventions or trial conduct	Pages 5, 6 & 7, Table 2
Auditing	23	Frequency and procedures for auditing trial conduct, if any, and whether the process will be independent from investigators and the sponsor	Page 8
**Ethics and dissemination**			
Research ethics approval	24	Plans for seeking research ethics committee/institutional review board (REC/IRB) approval	Page 9
Protocol amendments	25	Plans for communicating important protocol modifications (eg, changes to eligibility criteria, outcomes, analyses) to relevant parties (eg, investigators, REC/IRBs, trial participants, trial registries, journals, regulators)	Page 9
Consent or assent	26a	Who will obtain informed consent or assent from potential trial participants or authorised surrogates, and how (see Item 32)	Page 9
	26b	Additional consent provisions for collection and use of participant data and biological specimens in ancillary studies, if applicable	Page 9
Confidentiality	27	How personal information about potential and enrolled participants will be collected, shared, and maintained in order to protect confidentiality before, during, and after the trial	Page 9
Declaration of interests	28	Financial and other competing interests for principal investigators for the overall trial and each study site	Page 11
Access to data	29	Statement of who will have access to the final trial dataset, and disclosure of contractual agreements that limit such access for investigators	Page 9
Ancillary and post-trial care	30	Provisions, if any, for ancillary and post-trial care, and for compensation to those who suffer harm from trial participation	Page 9

For peer review only

Dissemination policy	31a	Plans for investigators and sponsor to communicate trial results to participants, healthcare professionals, the public, and other relevant groups (eg, via publication, reporting in results databases, or other data sharing arrangements), including any publication restrictions	Page 9
	31b	Authorship eligibility guidelines and any intended use of professional writers	Page 9
	31c	Plans, if any, for granting public access to the full protocol, participant-level dataset, and statistical code	Page 9
**Appendices**			
Informed consent materials	32	Model consent form and other related documentation given to participants and authorised surrogates	Appendix 2
Biological specimens	33	Plans for collection, laboratory evaluation, and storage of biological specimens for genetic or molecular analysis in the current trial and for future use in ancillary studies, if applicable	Not applicable

*It is strongly recommended that this checklist be read in conjunction with the SPIRIT 2013 Explanation & Elaboration for important clarification on the items. Amendments to the protocol should be tracked and dated. The SPIRIT checklist is copyrighted by the SPIRIT Group under the Creative Commons "Attribution-NonCommercial-NoDerivs 3.0 Unported" license.

For peer review only

BMJ Open

Best Emollients for Eczema (BEE) – comparing four types of emollients in children with eczema: protocol for randomised trial and nested qualitative study

Journal:	*BMJ Open*
Manuscript ID	bmjopen-2019-033387.R1
Article Type:	Protocol
Date Submitted by the Author:	03-Sep-2019
Complete List of Authors:	Ridd, Matthew; University of Bristol Faculty of Health Sciences, Population Health Sciences<br>Wells, Sian; University of Bristol, School of Social & Community Medicine<br>Edwards, Louisa; Simon Fraser University, Faculty of Health Sciences; Centre for Clinical Epidemiology and Evaluation (C2E2),<br>Santer, Miriam; University of Southampton, Primary Care & Population Sciences<br>MacNeill , Stephanie; University of Bristol, Bristol Randomised Trials Collaboration, Bristol Trials Centre<br>Sanderson, Emily; University of Bristol, Bristol Randomised Trials Collaboration, Bristol Trials Centre<br>Sutton, Eileen; University of Bristol, Population Health Sciences; University of Bristol<br>Shaw, Ali; University of Bristol, School of Social and Community Medicine<br>Banks, Jonathan; University of Bristol, Social & Community Medicine<br>Garfield, Kirsty; University of Bristol, Bristol Randomised Trials Collaboration<br>Roberts, Amanda; University of Nottingham, Centre of Evidence-Based Dermatology<br>Barrett, Tiffany; University Hospitals Bristol NHS Foundation Trust, South West Medicines Information & Training<br>Baxter, Helen; University of Bristol, Bristol Medical School: Population Health Sciences; University of Bristol<br>Taylor, Jodi; University of Bristol, School of Clinical Sciences<br>Lane, Athene; University of Bristol, School of Social and Community Medicine<br>Hay, Alastair; University of Bristol, Centre for Academic Primary Care<br>Williams, HC; University of Nottingham,<br>Thomas, Kim; University of Nottingham, Dermatology
**Primary Subject Heading**:	Dermatology
Secondary Subject Heading:	Paediatrics, Pharmacology and therapeutics, General practice / Family practice
Keywords:	Eczema < DERMATOLOGY, Paediatric dermatology < DERMATOLOGY, THERAPEUTICS

SCHOLARONE™
Manuscripts

Title page

Title

Best Emollients for Eczema (BEE) – comparing four types of emollients in children with eczema: protocol for randomised trial and nested qualitative study

Authors

Matthew J Ridd,[1] Sian Wells,[1] Louisa Edwards,[2] Miriam Santer,[3] Stephanie J MacNeill,[4] Emily Sanderson,[4] Eileen Sutton,[1] Alison R G Shaw,[1] Jonathan Banks,[6] Kirsty Garfield,[4] Amanda Roberts,[7] Tiffany J Barrett,[8] Helen Baxter,[1] Jodi Taylor,[4] J Athene Lane,[4] Alastair D Hay,[1] Hywel C Williams,[5] Kim Thomas[5]

[1] Centre for Academic Primary Care, Bristol Medical School, University of Bristol, UK.

[2] Faculty of Health Sciences, Simon Fraser University, Burnaby, Canada.

[3] Primary Care and Population Sciences, University of Southampton, Southampton, UK

[4] Bristol Randomised Trials Collaboration, Bristol Trials Centre, University of Bristol, Bristol, UK.

[5] Centre of Evidence Based Dermatology, University of Nottingham, Nottingham, UK

[6] National Institute for Health Research Collaborations for Leadership in Applied Health Research and Care West (NIHR CLAHRC West), University Hospitals Bristol NHS Foundation Trust, Bristol, UK

[7] Nottingham Support Group for Carers of Children with Eczema, Nottingham, UK.

[8] South West Medicines Information & Training, University Hospitals Bristol NHS Foundation Trust, Bristol, UK

Corresponding author

Matthew Ridd, GP and Reader in Primary Healthcare

Postal address: Centre for Academic Primary Care, Population Health Sciences, Bristol Medical School, University of Bristol, Canynge Hall, 39 Whatley Road, Bristol BS8 2PS

Email: m.ridd@bristol.ac.uk

Telephone: 0117 331 4557

Word count

Excluding title page, abstract, statements, references, box, tables and figure: 3815

Keywords

Atopic eczema/dermatitis, food allergy, feasibility RCT

For peer review only - http://bmjopen.bmj.com/site/about/guidelines.xhtml

For peer review only

For peer review only - http://bmjopen.bmj.com/site/about/guidelines.xhtml

ABSTRACT

Introduction: Atopic dermatitis/eczema affects around 20% of children and is characterised by inflamed, dry, itchy skin. Guidelines recommend "leave on" emollients that are applied directly to the skin to add or trap moisture and used regularly, they can soothe, enhance the skin barrier, and may prevent disease "flares". However, the suitability of the many different emollients varies between people and there is little evidence to help prescribers and parents and carers decide which type to try first.

Methods and analysis: Design: pragmatic, multi-centre, individually randomised, parallel group superiority trial of four types of emollient (lotions, creams, gel or ointments). Setting: GP surgeries at three sites in England. Participants: children aged over 6 months and less than 12 years with mild to severe eczema and no known sensitivity to study emollients. Interventions: study-approved lotion, cream, gel or ointment as the only leave-on emollient for 16 weeks, with directions to apply twice daily and as required. Other treatments, such as topical corticosteroids, used as standard care. Follow-up: 52 weeks. Primary outcome: validated parent-reported eczema symptoms (POEM) measured weekly for 16 weeks. Secondary outcomes: eczema signs (EASI) by masked researcher, treatment use, parent satisfaction, adverse events, child and family quality of life (ADQoL, CHU-9D and DFI). Sample size: 520 participants (130 per group). Analysis: intention-to-treat using linear mixed models for repeated measures. Nested qualitative study: audio-recording of sample of baseline appointments and up to 60 interviews with participants at four and 16 weeks, interviews to be transcribed and analysed thematically.

Ethics and dissemination: Ethics approval granted by the NHS REC (South West - Central Bristol Research Ethics Committee 17/SW/0089). Findings will be presented at conferences, published in open-access peer-reviewed journals and the study website; and summaries shared with key stakeholders.

Trial registration: ISRCTN: ISRCTN84540529 (Date registered: 05/06/2017)

Word count: 286/300

ARTICLE SUMMARY

Strength and limitations of this study

- First, adequately powered head-to-head pragmatic trial of the four main types of emollient prescribed for the treatment of eczema in children, recruited from primary care, with long-term follow-up.
- The primary core outcome is a validated patient-reported measure (POEM) that captures symptoms of eczema that matter to patients, and weekly measures over the 16-weeks mean that all participants who complete at least one POEM post-baseline will be included in the analysis. Researchers undertaking assessments of eczema signs (secondary outcome) are masked to allocation and use validated core outcome (EASI).
- Parents and their clinicians are unmasked and therefore their assessment of both the effectiveness and acceptability of the study emollient may be biased.
- Study emollients of each type are similar, increasing generalisability of the findings, but because they are not identical subtle differences both within and between-types may not be identified.

For peer review only - http://bmjopen.bmj.com/site/about/guidelines.xhtml

- The findings will reduce "trial-and-error" prescribing of initial choice of emollient but should not be used to restrict emollient options.

Main text

INTRODUCTION

Background and rationale

Eczema affects around 20% of children.[1] It is characterised by dry and inflamed itchy skin, and it can have a significant impact on the quality of life for both the child and their family.[2] In accordance with the recommended nomenclature of the World Allergy Organisation, we use the label "eczema" to refer to the clinical phenotype of atopic eczema/dermatitis.[3]

The majority of children with eczema have disease of mild or moderate severity and are diagnosed and managed exclusively in primary care.[4] Children are commonly prescribed a moisturiser (emollient) and topical corticosteroid/topical calcineurin inhibitor to use alongside to treat or prevent "flares".[5] By direct application to the skin, emollients improve skin hydration and reduce symptoms such as stinging or itching, but they can also act as a barrier to potential irritants. Mild anti-inflammatory properties may reduce reliance on topical corticosteroids/calcineurin inhibitors.[6] Many directly applied or "leave-on" emollients can also be used as soap substitutes.

However, there are many different emollients available and little evidence that any one emollient is better than another as a leave on treatment. The main formulations are lotions, creams, gels and ointments, which vary in their consistency from "light" to "heavy". This mainly reflects differences in their oil (lipid) to water ratios. Some products also contain humectants which help retain moisture, but emollients containing urea or antimicrobial compounds tend to be reserved for more severe disease.

The absence of evidence regarding the comparative clinical and cost-effectiveness of different products is reflected in emollient formularies. Clinician prescribing in the NHS is guided by locally produced and maintained formularies, which recommend which items should be prescribed in that area. In 2018, across England and Wales there were over 100 different emollient formularies which made widely varying recommendations about 109 different emollients.[7] The current situation where healthcare professionals recommend different emollients and carers find an effective emollient through a process of "trial and error" is detrimental to both families and the NHS.[8 9]

In 2007, NICE recommended research to identify "the most effective and cost-effective combinations of emollient products to use for the treatment of childhood atopic eczema".[5] A recently published Cochrane review identified 77 trials, comprising 6603 participants, evaluating the effectiveness of emollients.[6] The authors were unable to conclude whether some of the moisturisers, or their ingredients, are better than others, and recommended head to head comparisons in clinical trials.

Aim and objectives

The aim of the study is to compare the effectiveness and acceptability of four types of emollient (lotion, cream, gel and ointment) most commonly used to treat eczema.

The objectives are to compare the four different emollient types, over the medium (16 weeks) and long-term (52 weeks), with respect to:

For peer review only - http://bmjopen.bmj.com/site/about/guidelines.xhtml

- Parent-reported eczema symptoms
- Researcher assessment of eczema signs
- Quality of life for the child
- Impact of eczema on the family
- Adverse effects
- Acceptability of and parent satisfaction with study emollient
- Frequency and quantity of study emollient and other emollient use
- Use of other eczema treatments (including topical corticosteroid and calcineurin inhibitor)
- Number of well-controlled weeks

Trial design

BEE is a pragmatic, multi-centre, individually randomised, parallel group superiority trial of four types of emollient in children with eczema, with nested qualitative study.

It is a type A Clinical Trial of an Investigational Medicinal Product (CTIMP) trial, which is low risk because the use of the medicinal product is not higher than the risk of standard medical care.

METHODS AND ANALYSIS

Study setting

Primary care (GP surgeries) in and around Bristol, Southampton and Nottingham

Recruitment

The stages of participant recruitment are shown in Figure 1.

We will identify children aged between 6 months and less than 12 years with eczema via an electronic medical records search. A GP or a delegated member of the practice team will screen the search results for inclusion/exclusion criteria. Parents and carers (hereafter parents) of potentially eligible children will be posted an invitation. In addition, GPs can recruit participants opportunistically.

Interested parents will complete a brief screening questionnaire that will initially assess eligibility. Potentially eligible participants will be contacted by a member of the research team to explain more about the study and schedule a baseline appointment at which consent will be received.

Eligibility and allocation

Inclusion and exclusion criteria are summarised in the Box.

Participants will be randomised in a 1:1:1:1 ratio to the four groups, stratified by centre and minimised by baseline Patient Orientated Eczema Measure (POEM – mild 3-7, versus moderate/severe 8+)[10] and participant age (less than 2 years old versus 2 years and above) using a validated web-based randomisation system supplied by the Bristol Randomised Trials Collaboration. Allocation is secure, concealed and cannot be changed once made.

Intervention

In the NHS, GP prescribing is restricted by local formularies which vary widely and change over time. Therefore, participants will be randomised to a type of emollient (lotion, cream, gel or ointment) rather than a specific named emollient. However, to reduce heterogeneity within each type of emollient, GPs will be asked to only prescribe emollients which share certain characteristics (Table

For peer review only - http://bmjopen.bmj.com/site/about/guidelines.xhtml

1). Study emollients will therefore be distinct between types and similar within each type. It would be considered unethical to withhold an emollient from a participant, and so there is no "control" group.

At the baseline visit, the researcher will give parents simple verbal advice and a one-page summary on emollient use. GPs will issue a prescription of the study emollient with directions to "Use twice daily and as required" and make it available for repeat prescription. This is consistent with usual care, where clinician advice usually does not extend beyond what is written on the prescription, sometimes backed-up with an information leaflet. Parents will be contacted within one week of randomisation to ensure that they have collected and started using the study emollient. The amount of emollient used during the study will be determined by the family.

Parents will be asked to agree to use the study emollient as the only leave-on emollient for 16 weeks. However, if the family have problems with or dislike their study emollient, they can stop it and seek an alternative from their GP. In this instance, the GP/family will be encouraged to try another emollient of the same type.

Clinical management of eczema will otherwise be as usual, with participants free to continue using or change other treatments. Use of other emollients as soap substitutes for washing only is permissible and will not be classed as contamination.

Outcomes

The primary outcome is the POEM, measured weekly for 16 weeks. POEM is a patient-reported outcome that can be completed by proxy (carer report) and captures symptoms of importance to parents and patients over the previous week.[11] It demonstrates good validity, repeatability and responsiveness to change.[12 13] We have chosen repeated measures because eczema is a relapsing and remitting long-term condition and this approach captures effectiveness of treatments better than comparing outcomes at a single time point.

Secondary outcomes include:

- Eczema Area Severity Index (EASI)
- Use of study emollient/other eczema treatments
- Parent-reported satisfaction with study emollient
- Adverse events: localised reactions, slips and falls
- Child and family-oriented quality of life measures: Atopic Dermatitis Quality of Life (ADQoL);[14] Dermatitis Family Impact questionnaire (DFI)[15] and Child Health Utility 9D (CHU-9D),[16 17]

A complete schedule of data collection can be found in Table 2. We are following-up participants for one year because eczema is a relapsing-remitting condition where symptoms can be seasonal and there is paucity of long-term outcome data in relation to emollient use in children with eczema.

Participant timeline, data collection methods and participant retention

Participants will take part in the trial for 52 weeks, with the primary outcome collected over the first 16 weeks (Figure 1).

Baseline data will be collected by the researcher using paper case report forms (CRFs). Parents will be given the option of completing follow-up questionnaires either online or on paper. Parents are asked to complete weekly surveys for the first 16 weeks and then every 4 weeks between 16 and 52

For peer review only - http://bmjopen.bmj.com/site/about/guidelines.xhtml

weeks. With consent, participants' electronic medical records will be reviewed for data on prescriptions and consultations.

Parents will be sent regular newsletters and receive automatic emails or text reminders when their questionnaires are due. In recognition of their time and to encourage retention, parents will be offered £10 vouchers at the baseline and 16 weeks. We will also offer the child a small gift, e.g. "bee" toy, of about £5 in value.

Masking

Table 3 summarises who is masked to treatment allocation. Procedures to maintain masking to allocation will be written and followed. Researcher masking will be assessed using the Bang's blinding index.[18] Because parents, participants and treating clinicians will know the treatment allocation, un-masking procedures are not required.

Sample size

As we have four groups, we powered our sample size calculation to detect a clinically meaningful differences in six pairwise comparisons subsequent to a global test. We estimate that 416 participants (104 in each group) are required to detect a difference of 3.0 in POEM scores[12 19 20] between any two groups with 90% power and a significance level of 0.05 (after adjustment for multiple pairwise comparisons). We assumed a standard deviation (SD) of 5.5 (SD of 4.89 observed in feasibility trial[21]) to allow for greater variability in the data or smaller differences to be detected. To allow for 20% loss to follow-up, we propose recruiting 520 patients in total.

Data management

Personal data of participants' and their parents will be treated as strictly confidential and entered onto a secure administrative database stored on the University of Bristol server. Anonymised trial data will be collected and managed using the study's REDCap database.[22] This system will also be used to administer online questionnaires for those who choose for online rather than paper questionnaires. The system incorporates data entry and validation rules to reduce data entry errors and management functions to facilitate auditing and data quality assurance.

Statistical methods

The analysis and presentation of the trial data will be in accordance with Consolidated Standards of Reporting Trials (CONSORT) guidelines.[23 24] A full statistical analysis plan has been developed and approved by the independent statistician on the study's trial steering committee ahead of analysis of post-randomisation data and will be made available via the study website.

Baseline characteristics of patients will be compared between the four arms by reporting summary statistics. Characteristics will be reported as means and SD, medians and inter-quartile ranges or frequencies and proportions depending on the nature of the data and its distribution. If baseline characteristics of any two treatment groups differ by more than 10% or 0.5 SD then the effect of this variable on the primary outcome will be investigated in a sensitivity analysis.

Primary statistical analyses between the randomised groups will be conducted on an intention-to-treat (ITT) basis. For the primary outcome we will use linear mixed models (weekly observations, level 1, nested within participants, level 2) to explore whether there are differences in mean POEM scores between treatment groups after adjusting for baseline scores and all stratification and minimisation variables used in the randomisation. Pairwise comparisons will be conducted to

For peer review only - http://bmjopen.bmj.com/site/about/guidelines.xhtml

identify which intervention groups differed. To account for multiple testing, we will use a modified alpha of 0.0083 (0.05/6 pairwise comparisons equivalent).

Secondary outcomes will be analysed according to the data type and frequency of recording. Continuous outcomes measured at multiple time points will be analysed similarly to the primary outcome as described above. Continuous outcomes measured once after randomisation – such as EASI score – will be analysed using linear regression adjusting for baseline values where available. We will consider alternative methods should assumptions not be met.

To assess adherence to the allocated medication, for each participant, we will count the number of days of self-reported use of the allocated type of emollient and express that as a proportion of the number of days for which non-missing emollient data are available. Contamination will be assessed by calculating the proportion of days (among days where non-missing emollient data are available) where a non-allocated emollient type was used. We are unable to pre-specify what constitutes “substantial contamination”, which may inform further sensitivity analyses.

Other proposed sensitivity analyses include an exploration of patterns of missing data and we will consider possible mechanisms for this. Based on these and observed data, appropriate methods for imputing missing data will be considered in sensitivity analyses. Also, should there be evidence of imbalance between treatment groups on important baseline characteristics we will conduct a regression analysis of the primary outcome adjusting additionally for these variables.

Descriptive analysis of safety endpoints will be presented both according to randomised group. Pre-specified subgroup analyses will investigate whether treatment effectiveness is modified by the following factors measured at randomisation: parent expectation; age of child at randomisation; disease severity; and eczema diagnosis. These subgroup analyses will involve incorporating interaction terms with treatment allocation to test the null hypothesis of no variation in treatment effect across subgroups. These tests are likely to be underpowered, however, therefore emphasis will be placed on the point estimates and confidence intervals generated.

Nested qualitative study

The aims of the qualitative study are firstly, to support and optimise participant recruitment and retention; and secondly, to complement, explain and aid understanding of the quantitative findings

Baseline appointment recordings: To meet the first aim, a sample of baseline appointments (at least one per recruiting researcher) will be audio-recorded and reviewed a qualitative researcher. Using a structured template, the interaction will be reviewed to ensure key information is relayed and parent understanding checked. Recommendations will be feedback individually to the relevant researcher and collectively (anonymised) to other recruiting researchers and the trial management group. Prior to the start of the baseline appointment, parents will be asked to give verbal consent for the recording, with written consent obtained at the end of the appointment.

Interviews with parents and trial participants: To meet the second aim, we will interview parents and, at their discretion, the participating children themselves, at four weeks and 16 weeks after randomisation. The design is cross-sectional, with different families interviewed at each time point. However, where particularly interesting issues emerge, we may speak to a family at both time points. Parents will indicate on the trial consent form whether they are willing to be approached for these.

For peer review only - http://bmjopen.bmj.com/site/about/guidelines.xhtml

The four-week interviews will focus on the initial use and acceptability of the assigned emollient. We will conduct up to five interviews in each trial group (total ~20), purposively sampling by: recruitment centre, age of child, eczema severity and allocated type of emollient. We will include those who have stopped using the allocated treatment or switched emollient.

The 16-week interviews will focus on the overall experience of using the assigned emollient, perceived effectiveness, planned future use of emollients and experience of taking part in the trial. The sampling criteria will be the same as for the four week interviews, with the additional criterion of intentions regarding future emollient use. We expect to achieve data saturation by conducting up to 10 interviews in each trial group (total ~40).

Interviews are expected to last between 30-60 minutes. Topic guides (including sub-topic guide for children) will be used but with flexibility to allow unanticipated issues to emerge and be further explored in later interviews. Interviews will be captured using an encrypted digital voice recorder, transcribed and anonymised to protect confidentiality.

The interview data will be analysed thematically, using a combination of deductive and inductive coding[25] and adapted techniques of constant comparison.[26] Analysis will be led by the qualitative researcher, with input from the qualitative co-applicants and trial management group. Data management and coding will be aided by use of NVivo software. Data will be compared within and across trial group, with attention to converging and diverging perspectives. The themes will be written up as interpretive summaries with illustrative verbatim quotes that represent the range of expressed views.

Monitoring, safety and audit

As the randomised treatments within this study do not differ from common usual clinical practice, risk-based monitoring will be implemented in line with a risk-assessment. Data on adverse events will be collected by parent self-report. No interim analyses are planned.

An independent Data Monitoring Committee has been established and terms of reference have been drawn up and agreed. The committee will meet at least annually, and its role is to safeguard the interests of the trial's participants, potential participants, investigators and sponsor; to assess the safety and efficacy of the trial's interventions, and to monitor the trial's overall conduct, and protect its validity and credibility.

The sponsor organisation is the University of Bristol. Adverse event reporting will be in accordance with local procedures.

The trial may be prematurely discontinued due to lack of recruitment or by the Sponsor, Chief Investigator, Regulatory Authority or Funder based on new safety information or for other reasons given by the Trial Steering Committee or Data Monitoring Committee, regulatory authority or ethics committee concerned.

PUBLIC AND PATIENT INVOLVEMENT

In 2013, the James Lind Alliance published the eczema research priorities for patients and healthcare professionals and "Which emollients are the most effective and safe in treating eczema?" emerged as one of the highest ranked uncertainties.[27]

Co-author AR is mother of children with eczema and a member of Nottingham Support Group for Carers of Children with Eczema. We have established a group of parents of children with eczema,

For peer review only - http://bmjopen.bmj.com/site/about/guidelines.xhtml

who helped develop the study and want to support our on-going work through meetings and email communication. A PPI member sits on the trial steering committee. We will use the internet and social media to promote wider patient engagement.

PPI has helped us to frame the research question around, “Which emollient to prescribe first?” for childhood eczema, acknowledging that individuals differ in their experiences of effectiveness and tolerability of different emollients. It has also gave us a clear steer that including a non-emollient group would be unacceptable to many families; favoured POEM as the primary outcome; and highlighted how emollient use may be a “trade off” between effectiveness and acceptability.

On-going PPI involvement has informed both qualitative and quantitative data collection and helps ensure that the study continues to focus on delivering clinically important outcomes that are meaningful to patients.[28]

ETHICS AND DISSEMINATION

Research ethics approval

The study has been reviewed by the Health Research Authority and given a favourable opinion by the NHS REC (South West - Central Bristol Research Ethics Committee 17/SW/0089).

Protocol amendments

Any amendments to the Protocol will be reported accordingly to the regulatory bodies, with a copy of the current protocol (version 6.0 currently) available for download from the study website. Amendments to date are listed in appendix 1.

Consent and assent

Written consent for taking part in the trial will be received by a researcher from the parent or guardian of the participant at their baseline appointment. For children approximately 7 years and older, the option of providing assent will be offered alongside parental consent (see appendix 2).

Confidentiality and access to data

The database and randomisation system will protect patient information in line with the data protection legislation. Trial staff will ensure that participants’ anonymity is maintained through protective and secure handling and storage of patient information at the lead centre. The Chief Investigator will have access to and act as custodian of the full dataset.

Ancillary and post-trial care

After the 16-week primary outcome period, participants will be free to change their emollient if they wish. Conversely, they will be able to continue with their allocated emollient after they have completed follow-up.

Dissemination and data sharing

A series of stakeholder meetings will raise study awareness amongst and share progress and findings with policy makers, voluntary groups, clinicians, patients, families. Study progress, outputs and a summary of findings will be made available via a study website and Twitter account; and summaries distributed to participating families and GP surgeries. Findings will be submitted for presentation at conferences and written up for publication in a peer-reviewed journal(s), which may include integration of the quantitative and qualitative findings. The International Committee of Medical

For peer review only

For peer review only - http://bmjopen.bmj.com/site/about/guidelines.xhtml

Journal Editors has criteria for authorship will be observed and no professional writers will be employed.

No later than three years after the completion of the study, we will deposit a deidentified data set in an appropriate data archive.

DISCUSSION

Factors that may influence patient preference for different types of emollient include disease severity, body site, cosmetic acceptability of the product, season/climate and packaging.[29] Cultural factors may also influence choice and use.[30] NICE recommends patients try different emollients in the clinic before choosing.[5] This approach is not practical in primary care, and even in specialist settings the range of emollients available to try can be arbitrary – restricted by local formularies and influenced of pharmaceutical companies. Therefore, most patients consulting in primary care are unaware of differences between emollients; and many primary care clinicians will be unable to advise on grounds other than consistency or simple unit cost.

Some emollients are decades old and it has not been in the interest of manufacturers to submit their products in a head-to-head comparison with others in a clinical trial. In BEE, we are independently evaluating in a pragmatic trial, using a validated patient-reported primary outcome, the effectiveness of the four types of emollients commonly prescribed for children with eczema. In accordance with the recommendations of HOME (Harmonising Outcome Measures in Eczema), POEM and EASI will be used to measure patient-reported symptoms and clinical signs, respectively.[31]

Participants are unmasked, so by knowing which emollient they're using may bias assessment of emollient effectiveness. However, we have chosen a patient-reported outcome as the primary outcome because symptoms of eczema are more important to families of than objective measures which are based on skin appearance.[11] We will minimise the potential for performance bias by ensuring that at the point of consent parents are willing to use any of the four emollients for the first 16 weeks. We will also measure at baseline parent opinion regarding the four different study emollients, and in a sub-group analysis explore whether reported effectiveness is linked to high/low prior expectations of effectiveness. The collection of an objective measure of eczema severity (EASI) by a masked researcher as a secondary outcome allows us to examine outcomes in relation to signs of eczema. Subject to additional funding, we plan to undertake a full economic evaluation to determine the cost-effectiveness of the four emollient types.

Recruitment started in January 2018 and follow-up of the last participant is scheduled by February 2021. Findings from the BEE study, comparing the clinical effectiveness and acceptability of commonly used different emollients, will provide evidence upon which clinicians and carers/patients can decide which emollient to try first. Our aim is not to reduce choice, but to reduce uncertainty and the consequences of "trial and error" prescribing. Smarter prescribing will help prescribers and carers gain "control" over the eczema more quickly, reduce frustration and inconvenience for families, and potentially produce cost savings to the NHS through cost-effective prescribing and fewer repeat consultations to change emollients.

For peer review only - http://bmjopen.bmj.com/site/about/guidelines.xhtml

STATEMENTS

Acknowledgements

The BEE study:

- is hosted by Bristol, North Somerset and South Gloucestershire (BNSSG) CCG Research and Evidence.
- was developed with support from UK Dermatology Clinical Trials Network (UK DCTN). The UK DCTN is grateful to the British Association of Dermatologists and the University of Nottingham for financial support of the Network.
- was designed and is being delivered in collaboration with the Bristol Randomised Trials Collaboration (BRTC), a UKCRC registered clinical trials unit which, as part of the Bristol Trials Centre, is in receipt of National Institute for Health Research CTU support funding.

The TMG would like to thank Dr Sandra Hollinghurst for her advice on the collection of data relevant to health economics; and Mr Paul Roy and Ms Rachel Avery of BNSSG CCG Research and Evidence for their support in the delivery of the trial and dissemination of findings.

We are grateful to the members of the following trial oversight committees:

- Trial steering committee: Professor Richard McManus (chair), Dr Ben Carter (medical statistician), Professor Joanne Protheroe, Dr Sariqa Wagley and Dr Andrew Moore.
- Data monitoring committee members: Dr John Ingram (chair), Dr Catriona Keerie (medical statistician), Dr Chin Whybrew.

Declaration of interests

No interests to declare.

Funding

The study is funded by National Institute for Health Research (NIHR) HTA programme (Ref: 15/130/07). MJR is funded by an NIHR Post-Doctoral Research Fellowship (PDF-2014-07-013). JB was supported by the National Institute for Health Research (NIHR) Collaboration for Leadership in Applied Health Research and Care West (CLAHRC West) at University Hospital Bristol NHS Foundation Trust. The views expressed in this publication are those of the authors and not necessarily those of the NHS, the National Institute for Health Research or the Department of Health and Social Care.

Author contributions

MJR conceived the study idea and developed the initial study design with KT, HCW, MS, SJM, JB, AR, ARGS and ADH. TJB, JT and AL helped further develop the initial proposal and LE/SW assisted with the study protocol. Specific advice on trial design and medical statistics was given by SJM, ESa and JT; on the nested qualitative study by ARGS, JB and ESu; on the collection of health economic data by KG; and regarding dissemination by HB. Led by MJR, all the authors contributed to the drafting of the study protocol and approved the final manuscript.

For peer review only - http://bmjopen.bmj.com/site/about/guidelines.xhtml

Study website and social media

www.bristol.ac.uk/bee-study and Twitter: @bee-study

REFERENCES

1. Williams HC. Atopic dermatitis: The epidemiology, causes and prevention of atopic eczema. Cambridge: Cambridge University Press 2000.
2. Ben-Gashir M, Seed P, Hay R. Quality of life and disease severity are correlated in children with atopic dermatitis. *BJD* 2004;150(2):284-90.
3. Johansson S, Bieber T, Dahl R, et al. Revised nomenclature for allergy for global use: Report of the Nomenclature Review Committee of the World Allergy Organization, October 2003. *J Allergy Clin Immunol* 2004;113:832-36.
4. Emerson RM, Williams HC, Allen BR. Severity distribution of atopic dermatitis in the community and its relationship to secondary referral. *BJD* 1998;139:73-76.
5. NICE. CG057: Atopic eczema in children - management of atopic eczema in children from birth up to the age of 12 years. London: RCOG Press, 2007.
6. Van Zuuren EJ, Fedorowicz Z, Christensen R, et al. Emollients and moisturisers for eczema. *Cochrane Database of Systematic Reviews* 2017(2) doi: 10.1002/14651858.CD012119.pub2.
7. Chan JP, Boyd G, Quinn PA, et al. Emollient prescribing formularies in England and Wales: a cross-sectional study. *BMJ Open* 2018;8(6) doi: 10.1136/bmjopen-2018-022009
8. Santer M, Burgess H, Yardley L, et al. Experiences of carers managing childhood eczema and their views on its treatment: a qualitative study. *British Journal of General Practice* 2012;62(597):e261-e67. doi: 10.3399/bjgp12X636083
9. Le Roux E, Powell K, Banks JP, et al. GPs' experiences of diagnosing and managing childhood eczema: a qualitative study in primary care. *BJGP* 2018;68(667):e73-e80. doi: 10.3399/bjgp18X694529
10. Charman C, Venn A, Ravenscroft J, et al. Translating Patient-Oriented Eczema Measure (POEM) scores into clinical practice by suggesting severity strata derived using anchor-based methods. *BJD* 2013;169(6):1326-32. doi: 10.1111/bjd.12590
11. Charman CR, Venn AJ, Williams HC. The Patient-Oriented Eczema Measure: Development and Initial Validation of a New Tool for Measuring Atopic Eczema Severity From the Patients' Perspective. *Archives of Dermatology* 2004;140(12):1513-19.
12. Gaunt DM, Metcalfe C, Ridd M. The Patient-Oriented Eczema Measure in young children: responsiveness and minimal clinically important difference. *Allergy* 2016;71:1620-25. doi: 10.1111/all.12942
13. Schmitt J, Langan S, Williams HC. What are the best outcome measurements for atopic eczema? A systematic review. *Journal of Allergy and Clinical Immunology* 2007;120(6):1389-98. doi: 10.1016/j.jaci.2007.08.011
14. Stevens KJ, Brazier JE, McKenna SP, et al. The development of a preference-based measure of health in children with atopic dermatitis. *BJD* 2005;153(2):372-77.
15. Lawson V, Lewis-Jones MS, Finlay AY, et al. The family impact of childhood atopic dermatitis: the Dermatitis Family Impact Questionnaire. *BJD* 1998;138(1):107-13. doi: 10.1046/j.1365-2133.1998.02034.x
16. Stevens K. Assessing the performance of a new generic measure of health-related quality of life for children and refining it for use in health state valuation. *Applied Health Economics and Health Policy* 2011;9(3):157-69.
17. Stevens K, Katherine S. Valuation of the Child Health Utility 9D Index. *Pharmacoeconomics* 2012;30:729-47.
18. Bang H, Ni L, Davis CE. Assessment of blinding in clinical trials. *Controlled Clinical Trials* 2004;25(2):143-56. doi: http://dx.doi.org/10.1016/j.cct.2003.10.016

For peer review only - http://bmjopen.bmj.com/site/about/guidelines.xhtml

19. Schram ME, Spuls P, Leeflang MMG, et al. EASI, (objective) SCORAD and POEM for atopic eczema: responsiveness and minimal clinically important difference. *Allergy* 2012;67(1):99-106.
20. Howells L, Ratib S, Chalmers J R, Bradshaw L, Thomas K. How should minimally important change scores for the Patient-Oriented Eczema Measure be interpreted? A validation using varied methods. *BJD* 2018;178(5):1135-42. doi: doi:10.1111/bjd.16367
21. Ridd MJ, Garfield K, Gaunt DM, et al. Choice of Moisturiser for Eczema Treatment (COMET): feasibility study of a randomised controlled parallel group trial in children recruited from primary care. *BMJ Open* 2016;6(11) doi: 10.1136/bmjopen-2016-012021
22. Harris PA, Taylor R, Thielke R, et al. Research electronic data capture (REDCap)—A metadata-driven methodology and workflow process for providing translational research informatics support. *Journal of Biomedical Informatics* 2009;42(2):377-81. doi: https://doi.org/10.1016/j.jbi.2008.08.010
23. Calvert M, Blazeby J, Altman DG, et al. Reporting of patient-reported outcomes in randomized trials: The consort pro extension. *JAMA* 2013;309(8):814-22. doi: 10.1001/jama.2013.879
24. Zwarenstein M, Treweek S, Gagnier JJ, et al. Improving the reporting of pragmatic trials: an extension of the CONSORT statement. *BMJ* 2008;337 doi: 10.1136/bmj.a2390
25. Braun V, Clarke V. Using thematic analysis in psychology. *Qual Res Psychol* 2011;3 doi: 10.1191/1478088706qp063oa
26. Strauss AL, Corbin J. Basic of qualitative research: grounded theory procedures and techniques. London: Sage 1998.
27. Batchelor JM, Ridd MJ, Clarke T, et al. The Eczema Priority Setting Partnership: a collaboration between patients, carers, clinicians and researchers to identify and prioritize important research questions for the treatment of eczema. *BJD* 2013;168(3):577-82. doi: 10.1111/bjd.12040
28. Sutton E, Dawson S, Roberts A, et al. Patient and public involvement (PPI) in the Best Emollient for Eczema (BEE) Study. South West Meeting of the Society for Academic Primary Care. Southampton, 2019.
29. Moncrieff G. Use of emollients in dry skin conditions: consensus statement. *Clin Exp Dermatol* 2013;38:231-38.
30. Santer M, Muller I, Yardley L, et al. Parents' and carers' views about emollients for childhood eczema: qualitative interview study. *BMJ Open* 2016;6(8) doi: 10.1136/bmjopen-2016-011887
31. Schmitt J, Spuls P, Boers M, et al. Towards global consensus on outcome measures for atopic eczema research: results of the HOME II meeting. *Allergy* 2012;67:1111-17. doi: 10.1111/j.1398-9995.2012.02874.x

For peer review only

For peer review only - http://bmjopen.bmj.com/site/about/guidelines.xhtml

BOX

Box: Participant eligibility criteria

Inclusion criteria

Children must:

- be aged between 6 months and less than 12 years of age
- have eczema diagnosed by an appropriately qualified healthcare professional (registered doctor, nurse or health visitor)
- mild eczema or worse (POEM score>2 within previous 28 days)

The person giving consent must:

- have parental responsibility for the participant
- be willing to use the randomly allocated emollient type as the only leave-on emollient for 16 weeks.

Exclusion criteria

Child:

- known sensitivity to study emollients or their constituents
- participating in another research study currently or in the last four months
- any other known adverse medical or social circumstance that would make invitation to the study inappropriate (as determined by GP practice staff)

The person giving consent:

- unable to give informed consent
- insufficient written English to complete outcome measures

For peer review only

For peer review only - http://bmjopen.bmj.com/site/about/guidelines.xhtml

TABLES and FIGURE legend

Table 1: Rules for exclusion/inclusion of different types of emollients

Type of emollient		Lotion	Cream	Gel	Ointment
Rules/group shared characteristics	Exclusion	Antimicrobials or urea			
	Inclusion	Paraffin-based			
		Glycerol containing only	No humectant or lanolin	Does not contain povidine	No additives
Example formulary emollients from each group†		Cetraben lotion, QV lotion and Diprobase lotion	Diprobase cream, Epimax cream, Aquamax cream, Zerobase cream and AproDerm cream	Doublebase gel, Isomol gel, Zerodouble gel, AproDerm gel and MyriBase gel	Diprobase ointment, Emulsifying ointment BP, White soft/Liquid paraffin 50/50 ointment, Paraffin White soft ointment and Paraffin Yellow soft ointment

† Membership will be monitored and may change over time, keeping within the inclusion and exclusion criteria for each group.

For peer review only

Table 2: Schedule of enrolment, interventions and assessments

	Study period																												Close-out
	Enrolment	Allocation	Post-allocation																										
	S	V_0	Participant questionnaires																V_1	Participant questionnaires									
Week		0	1	2	3	4	5	6	7	8	9	10	11	12	13	14	15	16	16	20	24	28	32	36	40	44	48	52	
Parent completed																													
Screening questionnaire	●																												
Opinion about emollients		●																											
POEM	●	●	●	●	●	●	●	●	●	●	●	●	●	●	●	●	●	●		●	●	●	●	●	●	●	●	●	
Eczema pain & bother		●				●				●				●				●											
Use of treatments for eczema		●	●	●	●	●	●	●	●	●	●	●	●	●	●	●	●	●		●	●	●	●	●	●	●	●	●	
Adverse events			●	●	●	●	●	●	●	●	●	●	●	●	●	●	●	●		●	●	●	●	●	●	●	●	●	
Consultations (non-EMR)						●				●				●				●		●	●	●	●	●	●	●	●	●	
Personal costs						●				●				●				●		●	●	●	●	●	●	●	●	●	
DFI		●																●										●	
ADQoL		●						●										●										●	
CHU-9D		●						●										●										●	
Satisfaction with emollient																		●											
Study experiences																												●	
Researcher administered																													
Demographics and history		●																											
UK diagnostic criteria for AD		●																											
EASI		●																	●										
EMR notes review																													●
**Nested qualitative study**																													
Audio-recording		○																											
Round one interviews				←	○	→																							
Round two interviews																			← ○ →										

● = all participants; ○ = sample of participants

S: screening stage (responses to written invitation letters and people responding to opportunistic invites); V_0 and V_1: research face-to-face baseline & follow-up visits

POEM: Patient Orientated Eczema Measure; † bother score, itch intensity, parent global assessment; ADQoL: Atopic Dermatitis Quality of Life; IDQoL: Infant Dermatitis Quality of Life; CHU-9D: Children's Health Utility 9D; GAD-7: Generalised Anxiety Disorder 7; EASI: Eczema Area Severity Index; AD: Atopic Dermatitis. EMR: Electronic Medical Record

For peer review only - http://bmjopen.bmj.com/site/about/guidelines.xhtml

Table 3: Masking to treatment allocation

Individual(s)	Status
Participating children, their parents and any treating clinician	Unmasked: the allocated emollient is prescribed by the participant's GP and issued by local pharmacy as in usual care
Clinical Trials Unit (CTU) database staff, Trial Coordinators and Trial Administrator and Qualitative RA	Unmasked: CTU staff will maintain the randomisation database. The trial coordinator/administrator will randomise participants and be the initial point of contact for all enquiries relating to issues with the emollients.
Qualitative team (Drs Sutton, Heawood and Banks)	Unmasked: Participants will be sampled based on emollient allocation/use and during the interviews the qualitative researcher will specifically ask about the different emollient types.
Junior statistician (Ms Sanderson)	Unmasked: The junior statistician was initially masked knowing only an anonymised code for the different treatment groups. After approval of the statistical analysis plan, she was unmasked to permit preparation and discussion of unmasked data with the data monitoring committee.
Trial Manager and Chief Investigator	Masked: The Trial Manager was masked prior to the writing of the statistical analysis plan but is unmasked on an individual participant basis, when required to undertake randomisations and deal with potential serious adverse events. The Chief Investigator will only be unmasked in the event of a serious adverse event.
Other Trial Management Group members: Dr MacNeill (senior statistician), Dr Santer & Prof Thomas (PIs), Ms Barrett (pharmacist), Dr Lane & Dr Taylor (CTU), Professors Hay & Williams (senior researchers), Ms Kirsty Garfield (Health economist), Dr Baxter (knowledge mobilisation), Mrs Roberts (PPI)	Masked: procedures will be put in place to maintain masking both within and outside of project meetings.
Researchers	Masked: masking of researchers undertaking baseline and 16-week visits will be monitored by means of self-report.

For peer review only

Figure 1: Overview of participant pathway through the study

For peer review only

For peer review only - http://bmjopen.bmj.com/site/about/guidelines.xhtml

For peer review only

For peer review only - http://bmjopen.bmj.com/site/about/guidelines.xhtml

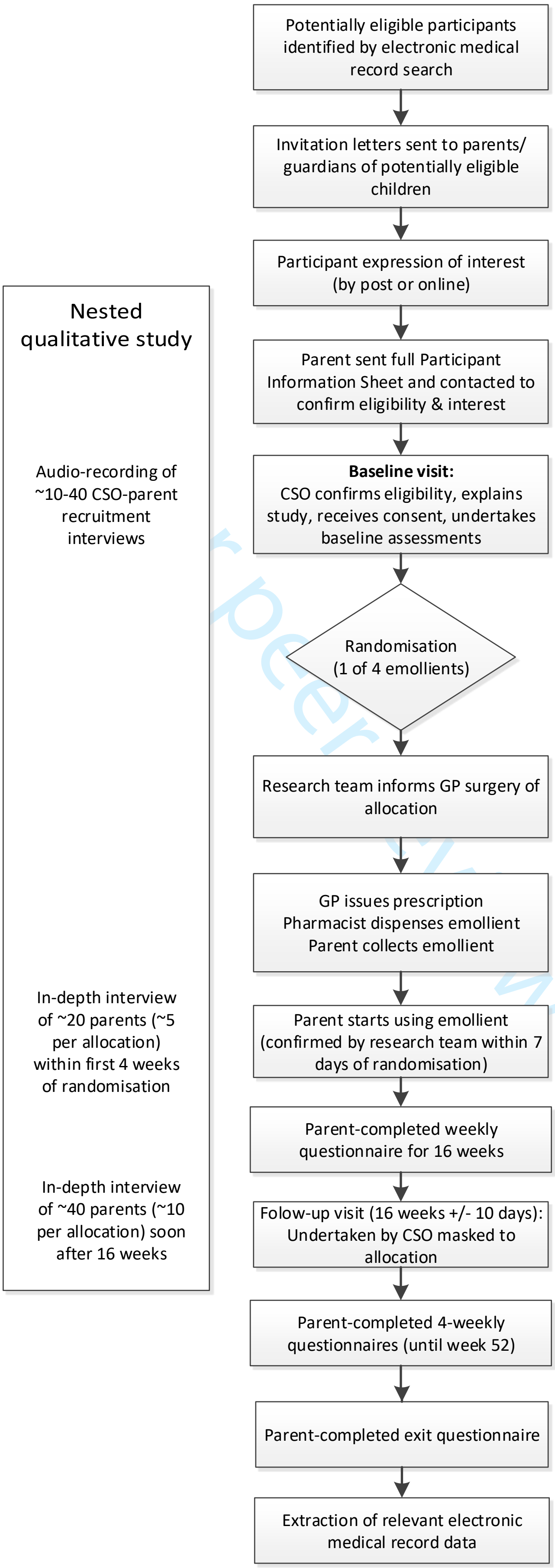
Potentially eligible participants identified by electronic medical record search
Invitation letters sent to parents/ guardians of potentially eligible children
Participant expression of interest (by post or online)
Parent sent full Participant Information Sheet and contacted to confirm eligibility & interest
Baseline visit: CSO confirms eligibility, explains study, receives consent, undertakes baseline assessments
Randomisation (1 of 4 emollients)
Research team informs GP surgery of allocation
GP issues prescription Pharmacist dispenses emollient Parent collects emollient
Parent starts using emollient (confirmed by research team within 7 days of randomisation)
Parent-completed weekly questionnaire for 16 weeks
Folow-up visit (16 weeks +/- 10 days): Undertaken by CSO masked to allocation
Parent-completed 4-weekly questionnaires (until week 52)
Parent-completed exit questionnaire
Extraction of relevant electronic medical record data
Nested qualitative study
Audio-recording of ~10-40 CSO-parent recruitment interviews
In-depth interview of ~20 parents (~5 per allocation) within first 4 weeks of randomisation
In-depth interview of ~40 parents (~10 per allocation) soon after 16 weeks

Appendix 1: Administrative information

Title

The Best Emollient for Eczema (BEE) trial: a randomised trial comparing the effectiveness of four types of commonly prescribed emollients for children with eczema

Trial registration number

ISRCTN: ISRCTN84540529 (Date registered: 05/06/2017)

World Health Organization Trial Registration Data Set

Data category	Information
Primary registry and trial identifying number	ISRCTN84540529
Date of registration in primary registry	05.06.2017
Secondary identifying numbers	EudraCT: 2017-000688-34
Source(s) of monetary or material support	NIHR Health Technology Assessment (HTA) 15/130/07
Primary sponsor	University of Bristol
Secondary sponsor(s)	Not applicable
Contact for public queries	bee-study@bristol.ac.uk, 0117 928 7351
Contact for scientific queries	Dr Matthew Ridd FRCGP PhD, m.ridd@bristol.ac.uk, 0117 331 4557
Public title	Best Emollients for Eczema (BEE) study
Scientific title	The Best Emollient for Eczema (BEE) trial: a randomised trial comparing the effectiveness of four types of commonly prescribed emollients for children with eczema
Countries of recruitment	England
Health condition(s) or problem(s) studied	Childhood eczema
Intervention(s)	Lotion, cream, gel or ointment as the only leave-on emollient for 16 weeks with directions to apply twice daily and as required.
Key inclusion and exclusion criteria	CHILD<br>Inclusion criteria:<br>1. Aged between 6 months and less than 12 years of age<br>2. Have eczema diagnosed by an appropriately qualified healthcare professional (registered doctor, nurse or health visitor)<br>3. Have mild eczema or worse (POEM score>2)<br><br>Exclusion criteria:<br>1. Known sensitivity to study emollients or their constituents<br>2. Participating in another research study currently or in the last four months<br>3. Any other known adverse medical or social circumstance that would

For peer review only - http://bmjopen.bmj.com/site/about/guidelines.xhtml

	make invitation to the study inappropriate (as determined by GP practice staff)<br><br>PERSON GIVING CONSENT:<br>Inclusion criteria:<br>1. Have parental responsibility for the participant<br>2. Willing to use the randomly allocated emollient as the only leave-on emollient for 16 weeks.<br><br>Exclusion criteria:<br>1. Unable to give informed consent<br>2. Insufficient written English to complete outcome measures.
Study type	Intervention
Date of first enrolment	19 January 2018
Target sample size	520
Recruitment status	Recruiting
Primary outcome(s)	Eczema symptoms, measured using POEM over 16 weeks
Key secondary outcomes	Eczema symptoms, measured using POEM over 52 weeks; eczema signs, measured using EASI; eczema 'bother' score; itch intensity score; parent global assessment of eczema; other possible symptoms of food allergy; UK diagnostic criteria for atopic dermatitis; main carer anxiety, measured using GAD-7; diet of child and/or mother if child being breastfed by her; adverse events; child and family quality of life, measured using ADQoL, CHU-9D and IDQoL; satisfaction with trial processes, procedures and paperwork; health services utilisation; out-of-pocket expenses/time off work.

Protocol version and history of amendments

The current version of the protocol is 6.0 (10.06.19). Previous protocols and amendments are as below:

Version		Notes
**Number**	**Date**	
1.0	21.03.2017	Submitted for approval (March 2017) and approval received from REC, MHRA and HRA.
2.0	27.06.2017	Title page: ISRCTN, NHS REC, and NIHR portfolio numbers added; 10.3: Clarification of eligibility confirmation; 10.6: "Blinding to treatment allocation" table amended to reflect changes in research team/processes to minimize un-blinding of TMG members, in accordance with TS/DM-C recommendation; 12.3: Clarification that first set of interviews will be with participants during their first four weeks in the study, not during the first four weeks of the life of the trial itself; 19.2: clarity to TS/DM-C composition/roles; 14.3: clarification about who makes decisions regarding causality of adverse events/reactions.
3.0	03.08.2017	Clarification that screening POEM must be within 28 days of recruitment. Removal of signature page to separate document.
4.0	03.11.17	Amendment to the intervention, from 4 specific emollients, to type of emollient. Correction of minor typos. Clarification of Safety reporting

For peer review only - http://bmjopen.bmj.com/site/about/guidelines.xhtml

		section. Update to “Timetable and milestones” to reflect delayed start to internal pilot.
5.0	01.08.18	Change “Bristol CCG” to “Bristol, North Somerset and South Gloucestershire CCG” to reflect merger/name change (page 6); changes to blinding arrangements (**Error! Reference source not found.**) and removal of reference to “Ms Jameson”, former CAPC PPI&E coordinator who was never a TMG member and has subsequently left (page 27/28); update to section 19.2 (Oversight committees) to describe separate TSC and DMC created at request of funder after approval of protocol 4.0; other minor grammatical/style changes/corrections.
6.0	10.06.19	Updated references to timelines throughout to reflect 38-months recruitment and follow-up / 50-month total study duration. Insertion of paragraph on participant communication (section 10.8, Participant stipends and communication). Replace Avon Primary Care Research Collaboration logo with BNSSG CCG logo. Replace any reference to blind, blinded or blinding with masked or masking. Extra information for parents of study participants in order to bring study in-line with the EU General Data Protection Regulations 2018. Minor changes to titles/postal addresses.

Funding

NIHR Health Technology Assessment (HTA) 15/130/07

Contributorship

See main manuscript

Sponsor contact information

Trial sponsor: University of Bristol
Sponsor’s reference: 2738
Contact name: Dr Rachel Davies
Address: Research Enterprise Development, One Cathedral Square, Bristol BS1 5DD
Email: research-governance@bristol.ac.uk
Telephone: 0117 428 4011

Role of study sponsor and funder

The funder and sponsor had no role in the design of this study and will not have any role during its execution, analyses, interpretation of the data, or decision to submit results.

Committees

The study is hosted by BNSSG CCG, and will be delivered by the University of Bristol, in collaboration with partners at University of Nottingham, University of Southampton. The Universities of Nottingham and Southampton will be recruiting centres, with Professor Kim Thomas and Dr Miriam Santer as the Principal Investigators, respectively.

The Trial Management Group comprises all investigators, the trial manager, research and administrative staff, the trials unit and patient/public representative. Members will contribute to the trial in the following ways: trial design and methods; participant recruitment and trial conduct; trial management; trial logistics and cost management; economic evaluation; qualitative study statistical data analysis; and publication. The Trial Management Group will meet on a regular basis to

For peer review only - http://bmjopen.bmj.com/site/about/guidelines.xhtml

For peer review only

oversee the management of the trial. Meetings will be face-to-face with teleconference facilities for TMG members who are unable to be present.

This study was designed and is being delivered in collaboration with the Bristol Randomised Trials Collaboration, a UKCRC registered clinical trials unit which, as part of the Bristol Trials Centre, is in receipt of National Institute for Health Research Clinical Trials Unit support funding.

Because this is a low-risk trial, the funder originally agreed that the roles of both guiding the TMG and monitoring trial data will be undertaken by a single joint committee, the Trial Steering/Data Monitoring Committee. However, because of changes implemented in version 4.0 of the protocol, the funder requested that separate Trial Steering and Data Monitoring Committees be established.

The Trial Steering Committee will provide overall supervision of the trial on behalf of the funder. Terms of reference have been drawn up and agreed with members, which comprises four independent members: a chairperson, an academic, a biostatistician and a patient representative (parent of child with eczema). There is one additional non-independent member who is a qualitative researcher. Non-independent members will not have any voting rights. The Trial Steering Committee will meet at least four times over the course of the study, including one which will coincide with the end of the internal pilot and a final meeting, when analysis is almost complete and the final report is being prepared.

The Data Monitoring Committee will safeguard the interests of the trial's participants, potential participants, investigators and sponsor; to assess the safety and efficacy of the trial's interventions, and to monitor the trial's overall conduct, and protect its validity and credibility. Terms of reference have been drawn up and agreed with members, which comprises three independent members: a chairperson, a biostatistician and GP with specialist interest in dermatology. The Data Monitoring Committee will meet at least annually: only committee members and the junior statistician should be present in closed sessions; open sessions will be attended by those at the closed session, plus the CI and possibly representatives of the sponsor or funder, and a trial unit representative.

For peer review only

For peer review only - http://bmjopen.bmj.com/site/about/guidelines.xhtml

University of BRISTOL · UNIVERSITY OF Southampton · The University of Nottingham UNITED KINGDOM · CHINA · MALAYSIA · Bristol Randomised Trials Collaboration

Best Emollient for Eczema (BEE) Study

BEE Best Emollient for Eczema

Parent/Carer Consent Form

Initial box

1. I confirm that I have read and understand the Participant Information Sheet dated 03.11.2017 (version 3.0) for the above study. I have had the opportunity to consider the information, ask questions and have had these questions answered satisfactorily. ☐
2. I understand that participation is voluntary and that we are free to withdraw at any time without giving any reason, without my child's medical care or legal rights being affected. ☐
3. I understand that after the study ends, the data collected will be made "open data". I understand that this means the anonymised data will be publicly available and may be used for purposes not related to this study, and it will not be possible to identify me from these data. ☐
4. I understand that relevant sections of my child's medical notes and all information collected for this research may be reviewed by the study team, from regulatory authorities or from the NHS Trust for the purpose of ensuring that the research is conducted appropriately. I give permission for these individuals to access my child's records as appropriate. ☐
5. I give permission for researchers working on this study to have access to my child's medical records for the purposes of collecting information relevant to the aims of this study. ☐
6. I give consent for the data collected in this trial to be used in future ethically approved studies on the understanding that all information will continue to be kept securely and remain confidential. ☐
7. I give consent to be contacted by a member of the research team with a view to being interviewed about my experiences of emollients and taking part in BEE. I understand that I will be given more information first, I can decide later about taking part in this, I will be asked to give further consent for taking part in interviews and that I may not be contacted at all. ☐
8. *For those asked to take part in audio-recording of recruitment visit only:* I agree to have my recruitment visit audio-recorded, including anything my child may say. I agree to data from my audio-recorded interview being transferred to and retained by the Universities of Bristol, Southampton and Nottingham for training, teaching and research purposes, now and in the future. ☐
9. I agree for my child to take part in the above-named study. ☐

______________________ Name of Participant (Child)	______________ Participant ID

______________________ Name of Parent/Guardian	______________ Signature	______________ Date
______________________ Name of person receiving consent	______________ Signature	______________ Date

Funded by NHS National Institute for Health Research

The research was funded by the NIHR Health Technology Assessment (HTA) Programme. The views expressed are those of the author(s) and not necessarily those of the NHS, the NIHR or the Department of Health.

For peer review only - http://bmjopen.bmj.com/site/about/guidelines.xhtml

University of Bristol · University of Southampton · The University of Nottingham UNITED KINGDOM · CHINA · MALAYSIA · Bristol Randomised Trials Collaboration

Best Emollient for Eczema (BEE) Study

Assent Form for Children

(Assent means you are agreeing to join this study)

Please circle Yes or No for each question

1.	I have read the leaflet that explains about the BEE study.	Yes	No
2.	I have been able to ask questions about it.	Yes	No
3.	I understand what the study is all about.	Yes	No
4.	I understand that I do not have to take part if I do not want to.	Yes	No
5.	I can change my mind and I do not have to say why.	Yes	No
6.	I agree to take part in the study.	Yes	No

Name of Participant (Child)	Signature	Participant ID
Name of person receiving assent	Signature	Date

For peer review only

Funded by NHS National Institute for Health Research

The research was funded by the NIHR Health Technology Assessment (HTA) Programme. The views expressed are those of the author(s) and not necessarily those of the NHS, the NIHR or the Department of Health.

For peer review only - http://bmjopen.bmj.com/site/about/guidelines.xhtml

SPIRIT 2013 Checklist: Recommended items to address in a clinical trial protocol and related documents*

Section/item	ItemNo	Description	Location
**Administrative information**			
Title	1	Descriptive title identifying the study design, population, interventions, and, if applicable, trial acronym	Page 1
Trial registration	2a	Trial identifier and registry name. If not yet registered, name of intended registry	Page 2
	2b	All items from the World Health Organization Trial Registration Data Set	Appendix 1
Protocol version	3	Date and version identifier	Page 9 & Appendix 1
Funding	4	Sources and types of financial, material, and other support	Page 11
Roles and responsibilities	5a	Names, affiliations, and roles of protocol contributors	Page 1
	5b	Name and contact information for the trial sponsor	Appendix 1
	5c	Role of study sponsor and funders, if any, in study design; collection, management, analysis, and interpretation of data; writing of the report; and the decision to submit the report for publication, including whether they will have ultimate authority over any of these activities	Appendix 1
	5d	Composition, roles, and responsibilities of the coordinating centre, steering committee, endpoint adjudication committee, data management team, and other individuals or groups overseeing the trial, if applicable (see Item 21a for data monitoring committee)	Page 11 & Appendix 1
**Introduction**			

For peer review only

Background and rationale	6a	Description of research question and justification for undertaking the trial, including summary of relevant studies (published and unpublished) examining benefits and harms for each intervention	Page 3
	6b	Explanation for choice of comparators	Pages 3 & 4
Objectives	7	Specific objectives or hypotheses	Pages 3 & 4
Trial design	8	Description of trial design including type of trial (eg, parallel group, crossover, factorial, single group), allocation ratio, and framework (eg, superiority, equivalence, noninferiority, exploratory)	Page 4
**Methods: Participants, interventions, and outcomes**			
Study setting	9	Description of study settings (eg, community clinic, academic hospital) and list of countries where data will be collected. Reference to where list of study sites can be obtained	Page 4
Eligibility criteria	10	Inclusion and exclusion criteria for participants. If applicable, eligibility criteria for study centres and individuals who will perform the interventions (eg, surgeons, psychotherapists)	Page 4/Box
Interventions	11a	Interventions for each group with sufficient detail to allow replication, including how and when they will be administered	Pages 4 & 5, Table 1
	11b	Criteria for discontinuing or modifying allocated interventions for a given trial participant (eg, drug dose change in response to harms, participant request, or improving/worsening disease)	Page 5
	11c	Strategies to improve adherence to intervention protocols, and any procedures for monitoring adherence (eg, drug tablet return, laboratory tests)	Pages 5 & 7
	11d	Relevant concomitant care and interventions that are permitted or prohibited during the trial	Page 5

For peer review only

Outcomes	12	Primary, secondary, and other outcomes, including the specific measurement variable (eg, systolic blood pressure), analysis metric (eg, change from baseline, final value, time to event), method of aggregation (eg, median, proportion), and time point for each outcome. Explanation of the clinical relevance of chosen efficacy and harm outcomes is strongly recommended	Page 5 and Table 2
Participant timeline	13	Time schedule of enrolment, interventions (including any run-ins and washouts), assessments, and visits for participants. A schematic diagram is highly recommended (see Figure)	Page 5, Figure 1 & Table 2
Sample size	14	Estimated number of participants needed to achieve study objectives and how it was determined, including clinical and statistical assumptions supporting any sample size calculations	Page 6
Recruitment	15	Strategies for achieving adequate participant enrolment to reach target sample size	Pages 4 & 7
**Methods: Assignment of interventions (for controlled trials)**			
Allocation:			
Sequence generation	16a	Method of generating the allocation sequence (eg, computer-generated random numbers), and list of any factors for stratification. To reduce predictability of a random sequence, details of any planned restriction (eg, blocking) should be provided in a separate document that is unavailable to those who enrol participants or assign interventions	Page 4
Allocation concealment mechanism	16b	Mechanism of implementing the allocation sequence (eg, central telephone; sequentially numbered, opaque, sealed envelopes), describing any steps to conceal the sequence until interventions are assigned	Page 4 & Table 3
Implementation	16c	Who will generate the allocation sequence, who will enrol participants, and who will assign participants to interventions	Page 4, Figure 1, Table 3
Blinding (masking)	17a	Who will be blinded after assignment to interventions (eg, trial participants, care providers, outcome assessors, data analysts), and how	Page 6 & Table 3

For peer review only - http://bmjopen.bmj.com/site/about/guidelines.xhtml

	17b	If blinded, circumstances under which unblinding is permissible, and procedure for revealing a participant's allocated intervention during the trial	N/A
**Methods: Data collection, management, and analysis**			
Data collection methods	18a	Plans for assessment and collection of outcome, baseline, and other trial data, including any related processes to promote data quality (eg, duplicate measurements, training of assessors) and a description of study instruments (eg, questionnaires, laboratory tests) along with their reliability and validity, if known. Reference to where data collection forms can be found, if not in the protocol	Page 5, Table 2
	18b	Plans to promote participant retention and complete follow-up, including list of any outcome data to be collected for participants who discontinue or deviate from intervention protocols	Page 5
Data management	19	Plans for data entry, coding, security, and storage, including any related processes to promote data quality (eg, double data entry; range checks for data values). Reference to where details of data management procedures can be found, if not in the protocol	Page 6
Statistical methods	20a	Statistical methods for analysing primary and secondary outcomes. Reference to where other details of the statistical analysis plan can be found, if not in the protocol	Pages 6 & 7
	20b	Methods for any additional analyses (eg, subgroup and adjusted analyses)	Pages 6 & 7
	20c	Definition of analysis population relating to protocol non-adherence (eg, as randomised analysis), and any statistical methods to handle missing data (eg, multiple imputation)	Page 7
**Methods: Monitoring**			
Data monitoring	21a	Composition of data monitoring committee (DMC); summary of its role and reporting structure; statement of whether it is independent from the sponsor and competing interests; and reference to where further details about its charter can be found, if not in the protocol. Alternatively, an explanation of why a DMC is not needed	Pages 8 & 11, Appendix 1

	21b	Description of any interim analyses and stopping guidelines, including who will have access to these interim results and make the final decision to terminate the trial	Page 8 and Appendix 1
Harms	22	Plans for collecting, assessing, reporting, and managing solicited and spontaneously reported adverse events and other unintended effects of trial interventions or trial conduct	Pages 5, 6 & 7, Table 2
Auditing	23	Frequency and procedures for auditing trial conduct, if any, and whether the process will be independent from investigators and the sponsor	Page 8
**Ethics and dissemination**			
Research ethics approval	24	Plans for seeking research ethics committee/institutional review board (REC/IRB) approval	Page 9
Protocol amendments	25	Plans for communicating important protocol modifications (eg, changes to eligibility criteria, outcomes, analyses) to relevant parties (eg, investigators, REC/IRBs, trial participants, trial registries, journals, regulators)	Page 9
Consent or assent	26a	Who will obtain informed consent or assent from potential trial participants or authorised surrogates, and how (see Item 32)	Page 9
	26b	Additional consent provisions for collection and use of participant data and biological specimens in ancillary studies, if applicable	Page 9
Confidentiality	27	How personal information about potential and enrolled participants will be collected, shared, and maintained in order to protect confidentiality before, during, and after the trial	Page 9
Declaration of interests	28	Financial and other competing interests for principal investigators for the overall trial and each study site	Page 11
Access to data	29	Statement of who will have access to the final trial dataset, and disclosure of contractual agreements that limit such access for investigators	Page 9
Ancillary and post-trial care	30	Provisions, if any, for ancillary and post-trial care, and for compensation to those who suffer harm from trial participation	Page 9

For peer review only

Dissemination policy	31a	Plans for investigators and sponsor to communicate trial results to participants, healthcare professionals, the public, and other relevant groups (eg, via publication, reporting in results databases, or other data sharing arrangements), including any publication restrictions	Page 9
	31b	Authorship eligibility guidelines and any intended use of professional writers	Page 9
	31c	Plans, if any, for granting public access to the full protocol, participant-level dataset, and statistical code	Page 9
**Appendices**			
Informed consent materials	32	Model consent form and other related documentation given to participants and authorised surrogates	Appendix 2
Biological specimens	33	Plans for collection, laboratory evaluation, and storage of biological specimens for genetic or molecular analysis in the current trial and for future use in ancillary studies, if applicable	Not applicable

*It is strongly recommended that this checklist be read in conjunction with the SPIRIT 2013 Explanation & Elaboration for important clarification on the items. Amendments to the protocol should be tracked and dated. The SPIRIT checklist is copyrighted by the SPIRIT Group under the Creative Commons "Attribution-NonCommercial-NoDerivs 3.0 Unported" license.

For peer review only
