# Supplementary material for: Best emollients for eczema (BEE) – comparing four types of emollients in children with eczema: protocol for randomised trial and nested qualitative study
Source: BMJ Open. 2019 Nov 6;9(11):e033387. doi: 10.1136/bmjopen-2019-033387 (PMC6858146; doi:10.1136/bmjopen-2019-033387)
Supplement: Reviewer comments [file bmjopen-2019-033387.reviewer_comments.pdf]

PEER REVIEW HISTORY

BMJ Open publishes all reviews undertaken for accepted manuscripts. Reviewers are asked to complete a checklist review form (**http://bmjopen.bmj.com/site/about/resources/checklist.pdf**) and are provided with free text boxes to elaborate on their assessment. These free text comments are reproduced below.

ARTICLE DETAILS

**TITLE (PROVISIONAL)**	Best Emollients for Eczema (BEE) – comparing four types of emollients in children with eczema: protocol for randomised trial and nested qualitative study
**AUTHORS**	Ridd, Matthew; Wells, Sian; Edwards, Louisa; Santer, Miriam; MacNeill, Stephanie; Sanderson, Emily; Sutton, Eileen; Shaw, Ali; Banks, Jonathan; Garfield, Kirsty; Roberts, Amanda; Barrett, Tiffany; Baxter, Helen; Taylor, Jodi; Lane, Athene; Hay, Alastair; Williams, HC; Thomas, Kim

This article was not externally reviewed at BMJ Open. Protocols that have been independently assessed prior to submission to BMJ Open are usually fast-tracked to publication on the grounds that further substantial changes will not be possible. This independent assessment will usually be external, independent review for both a substantial grant award from a non-commercial or government funder and ethics approval.
